# Redox-mediated activation of ATG3 promotes ATG8 lipidation and autophagy progression in *Chlamydomonas reinhardtii*

**DOI:** 10.1093/plphys/kiad520

**Published:** 2023-09-29

**Authors:** Manuel J Mallén-Ponce, María Esther Pérez-Pérez

**Affiliations:** Instituto de Bioquímica Vegetal y Fotosíntesis (IBVF), Consejo Superior de Investigaciones Científicas (CSIC)- Universidad de Sevilla, Sevilla 41092, Spain; Instituto de Bioquímica Vegetal y Fotosíntesis (IBVF), Consejo Superior de Investigaciones Científicas (CSIC)- Universidad de Sevilla, Sevilla 41092, Spain

## Abstract

Autophagy is one of the main degradative pathways used by eukaryotic organisms to eliminate useless or damaged intracellular material to maintain cellular homeostasis under stress conditions. Mounting evidence indicates a strong interplay between the generation of reactive oxygen species and the activation of autophagy. Although a tight redox regulation of autophagy has been shown in several organisms, including microalgae, the molecular mechanisms underlying this control remain poorly understood. In this study, we have performed an in-depth in vitro and in vivo redox characterization of ATG3, an E2-activating enzyme involved in ATG8 lipidation and autophagosome formation, from 2 evolutionary distant unicellular model organisms: the green microalga *Chlamydomonas* (*Chlamydomonas reinhardtii*) and the budding yeast *Saccharomyces cerevisiae*. Our results indicated that ATG3 activity from both organisms is subjected to redox regulation since these proteins require reducing equivalents to transfer ATG8 to the phospholipid phosphatidylethanolamine. We established the catalytic Cys of ATG3 as a redox target in algal and yeast proteins and showed that the oxidoreductase thioredoxin efficiently reduces ATG3. Moreover, in vivo studies revealed that the redox state of ATG3 from *Chlamydomonas* undergoes profound changes under autophagy-activating stress conditions, such as the absence of photoprotective carotenoids, the inhibition of fatty acid synthesis, or high light irradiance. Thus, our results indicate that the redox-mediated activation of ATG3 regulates ATG8 lipidation under oxidative stress conditions in this model microalga.

## Teaser

The redox state of the ATG3 E2-activating enzyme plays a fundamental role in the control of ATG8 lipidation under reactive oxygen species–linked autophagy activation in the model green alga *Chlamydomonas reinhardtii*.

## Introduction

Macroautophagy (hereafter autophagy) is a dynamic and highly conserved recycling mechanism by which eukaryotic cells degrade cytoplasmic components, including protein aggregates or dysfunctional organelles in the vacuole (or lysosome). This catabolic pathway is an adaptive response to different stress conditions that allows cells to maintain cellular homeostasis and cope with stress (He and Klionsky 2009; Mizushima et al. 2011). Although basal autophagy plays a housekeeping function in the absence of stress, this degradative process is upregulated to eliminate unneeded cellular material under adverse conditions (Signorelli et al. 2019). Autophagy was initially considered as a bulk degradation pathway to provide building blocks and recycle nutrients, but it is well established that intracellular content can be specifically degraded through selective autophagy. Indeed, selective degradation of organelles and complex macromolecules such as mitochondria (mitophagy), chloroplast (chlorophagy), or proteasome (proteaphagy) has been reported (Kanki et al. 2009; Marshall et al. 2015; Galluzzi et al. 2017; Izumi et al. 2017; Gatica et al. 2018; Adriaenssens et al. 2022).

Autophagy is characterized by the de novo biogenesis of double-membrane vesicles or autophagosomes, which engulf and deliver cytoplasmic material or cargo to the vacuole for degradation and recycling (Supplemental Fig. S1). Thus, autophagosome formation is a morphological hallmark of autophagy and has been widely used to monitor autophagy activation in different organisms, including the model single-celled microalga *Chlamydomonas* (*Chlamydomonas reinhardtii*) (Kirisako et al. 1999; Yoshimoto et al. 2004; Pérez-Pérez et al. 2010).

Autophagy is carried out by more than 40 autophagy-related (ATG) proteins, most of which have been described in yeasts (Tsukada and Ohsumi 1993; Ichimura et al. 2000; Díaz-Troya et al. 2008; Liu and Bassham 2012; Shemi et al. 2015). Autophagosome biogenesis is mediated by a set of ATG proteins that constitute the core autophagy machinery (Xie and Klionsky 2007; Nakatogawa et al. 2009; Mizushima et al. 2011; Nakatogawa 2020). Among them, ATG8 plays a crucial function in autophagy and is the only known ATG protein that binds tightly to autophagic membranes throughout the whole autophagy process, from the recruitment of ATG proteins to the phagophore assembly site (PAS) to the release of the autophagic body into the vacuole. ATG8 plays a critical role in autophagosome formation and contributes to cargo recognition and autophagosome tethering to the vacuolar membrane (Kirisako et al. 1999, 2000; Ichimura et al. 2000).

To perform these functions, ATG8 must first bind to the phospholipid phosphatidylethanolamine (PE) to form the ATG8-PE adduct in a process known as ATG8 lipidation or ATG8 conjugation (Supplemental Fig. S1). The binding of ATG8 to PE occurs through a set of sequential and coordinated reactions governed by the ubiquitin-like ATG8 system (Ichimura et al. 2000; Kirisako et al. 2000). First, nascent ATG8 is processed at a highly conserved Gly at its C-terminus by the Cys-protease ATG4. Then, the E1-like enzyme ATG7 activates and transfers processed ATG8 to the catalytic Cys of the E2-like enzyme ATG3. Finally, ATG3 catalyzes the covalent binding of PE to the C-terminal Gly of ATG8 to form the ATG8-PE conjugate (Ichimura et al. 2000; Kirisako et al. 2000). The ATG12–ATG5 conjugate, formed by the ubiquitin-like ATG12 system, acts as an E3-like ligase that potentiates the final step of ATG8 lipidation by rearranging the catalytic site of ATG3 (Hanada et al. 2007; Sakoh-Nakatogawa et al. 2013). The ATG4 protease also has a deconjugating activity that cleaves ATG8-PE and releases free ATG8 from membranes (Kirisako et al. 2000) (Supplemental Fig. S1). Therefore, the activity of proteins from the ATG8-conjugating system controls the balance between free and lipidated ATG8, which is essential for autophagosome formation. Consequently, lipidated ATG8 has been widely used as an autophagy marker due to the relation between the formation of ATG8-PE and the induction of this catabolic process (Kilonsky et al. 2021a).

Autophagy has to be tightly regulated since loss of regulation of this catabolic process results in cellular dysfunction, hypersensitivity to stress, and metabolic disorders such as cancer and neurodegenerative diseases in humans (Hofius et al. 2009; Levine and Kroemer 2019; Signorelli et al. 2019; Kilonsky et al. 2021b). It has been demonstrated that the target of rapamycin (TOR) kinase, a master regulator of cell growth, negatively regulates autophagy in multiple organisms, ranging from unicellular yeasts and algae to multicellular plants and animals (Noda and Ohsumi 1998; Liu and Bassham 2010; Pérez-Pérez et al. 2010). In addition to TOR, the sucrose nonfermenting kinase 1 (Snf1)/AMP-activated protein kinase (AMPK)/Snf1-related kinase 1 (SnRK1) kinase activates autophagy in response to energy or nutrient deficiency (Soto-Burgos and Bassham 2017). On the other hand, mounting evidence indicates that the production of reactive oxygen species (ROS) under stress conditions regulates autophagy in different organisms. In *Chlamydomonas*, ROS participate in the activation of autophagy under oxidative stress generated by direct addition of H_2_O_2_ (Pérez-Pérez et al. 2010), methyl viologen (MV) treatment (Pérez-Pérez, Couso, and Crespo 2012), endoplasmic reticulum stress (Pérez-Pérez et al. 2010; Pérez-Martín et al. 2014), carotenoid depletion (Pérez-Pérez, Couso, and Crespo 2012), chloroplast damage (Heredia-Martínez et al. 2018), high light (HL) stress (Pérez-Pérez, Couso, and Crespo 2012; Kuo et al. 2020), or impaired starch biosynthesis (Tran et al. 2019). In close agreement, autophagy is involved in degrading oxidized proteins under oxidative stress conditions in *Arabidopsis* (*Arabidopsis thaliana*) (Xiong et al. 2007). Moreover, several studies in plants suggest that SnRK2 activates autophagy in response to ROS produced under abiotic and biotic stress conditions by inhibiting TOR kinase (Rosenberger and Chen 2018; Signorelli et al. 2019). Thus, an interplay between ROS and autophagy has been described in both plants and algae since ROS activates autophagy as a protective mechanism to decrease ROS production (Pérez-Pérez, Couso, and Crespo 2012; Signorelli et al. 2019).

The control of autophagy by redox signals is an emerging research field, and the molecular mechanisms underlying this regulation have not been fully elucidated. The activity of the Cys-protease ATG4 has been shown to be redox regulated. In mammals, ATG4 is a direct target of H_2_O_2_, and ROS produced in mitochondria serve as a signaling molecule in nutrient starvation–induced autophagy (Scherz-Shouval et al. 2007). Detailed biochemical studies carried out in yeasts and algae demonstrated that ATG4 is subjected to redox posttranslational modifications (PTMs) in these organisms (Pérez-Pérez et al. 2014, 2016). These studies also uncovered the basic mechanism for the redox regulation of yeast and algal ATG4 proteins, which involves the formation of a single disulfide bond with a very low redox potential that can be efficiently reduced by thioredoxin (Trx). It has also been shown that ATG4 oxidation leads to further ATG4 inactivation through oligomer formation in yeasts and algae (Pérez-Pérez et al. 2014, 2016, 2021). In consonance with the redox regulation of human, yeast, and algal ATG4 proteins, reversible inhibition of ATG4 by H_2_O_2_ has also been proposed in plants (Woo et al. 2014).

ATG4 does not seem to be the only ATG protein subject to redox regulation. Under oxidative stress, direct oxidation of human ATG3 and ATG7 at the active site thiols prevents lipidation of LC3 (an ATG8 isoform in mammals) and, thus, autophagy activation (Frudd et al. 2018). Whether redox regulation of human ATG3 is conserved in other organisms is unknown. In this study, we have performed an in-depth biochemical analysis of the ATG3 protein from *C. reinhardtii* and *Saccharomyces cerevisiae*, 2 evolutionary distant model organisms with highly conserved autophagy machinery. We have investigated the redox regulation of the ATG8-conjugating activity of ATG3 by in vitro and in vivo approaches. Our data unraveled the molecular mechanism by which redox signals control ATG3 activity to promote ATG8 lipidation and autophagy progression in *Chlamydomonas* subjected to oxidative damage generated by chloroplast damage.

## Results

### ATG3 regulation depends on the redox potential

**Figure 1.**
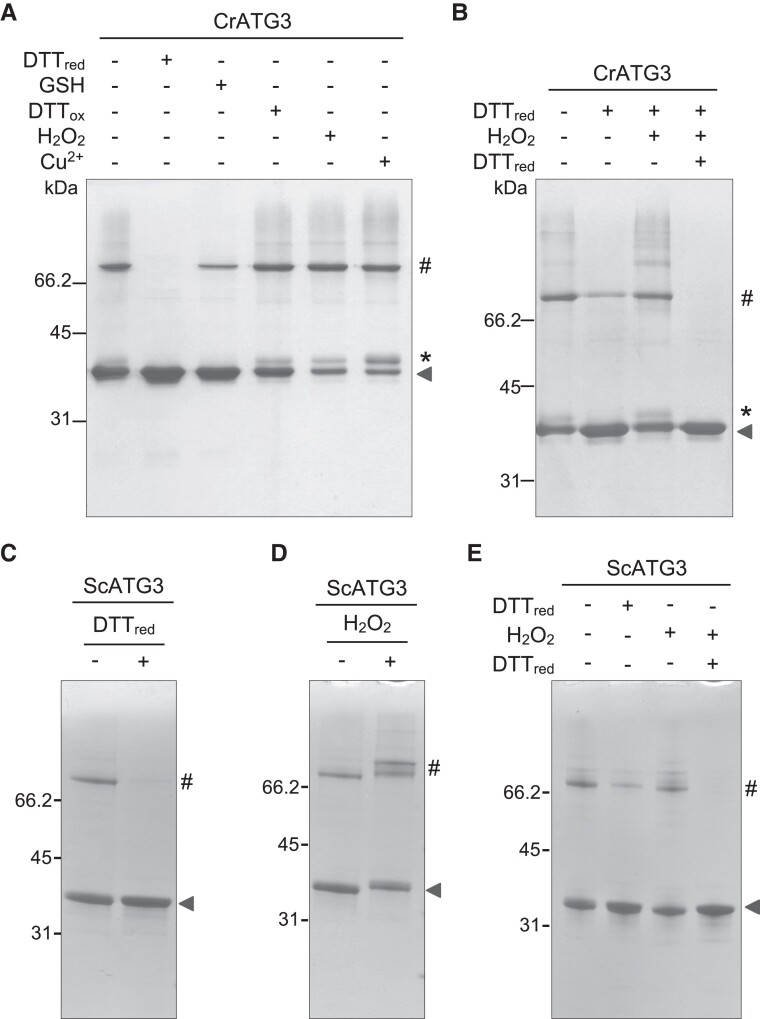
Reversible oxidation of ATG3 from *C. reinhardtii* and *S. cerevisiae.***A)***Chlamydomonas* ATG3 (CrATG3) untreated (−) or treated (+) with different redox agents such as DTT_red_ (2.5 mM), GSH (5 mM), DTT_ox_ (2.5 mM), H_2_O_2_ (0.5 mM), or Cu^2+^ (0.1 mM) for 30 min and then resolved by 12% nonreducing SDS–PAGE gel. **B)** CrATG3 untreated (−) or treated (+) with the reducer DTT_red_ (0.5 mM, 30 min), then with the oxidant H_2_O_2_ (1 mM, 20 min), and finally with a higher DTT_red_ concentration (10 mM, 30 min). **C)** ScATG3 untreated (−) or treated (+) with DTT_red_ (2.5 mM, 30 min). **D)** ScATG3 in the absence (−) or presence (+) of H_2_O_2_ (0.5 mM, 30 min). **E)** ScATG3 was sequentially treated (+) with 0.5 mM DTT for 30 min, then with 1 mM H_2_O_2_ for 20 min, and finally with 10 mM DTT for 30 min. All incubation assays were performed at 25 °C using 2.5 *µ*g of purified CrATG3 **A, B)** or ScATG3 **C to E)**. In all experiments, untreated sample (−) was used as control. The molecular mass marker (kDa) is shown on the left. The arrow indicates reduced monomeric CrATG3 or ScATG3. The symbols * and # indicate oxidized monomeric CrATG3 and dimeric CrATG3 or ScATG3, respectively.

In order to investigate whether the *C. reinhardtii* ATG3 (CrATG3) protein is subjected to redox regulation, we performed a detailed biochemical analysis of the recombinant protein under different reducing and oxidizing conditions. CrATG3 migrated as a 36-kDa protein in the presence of β-mercaptoethanol (βME); however, an additional band of ≍70 kDa, likely corresponding to dimeric CrATG3, was observed in nonreducing denaturing gels (Supplemental Fig. S2, A and B). Incubation of CrATG3 with reducing agents such as DTT_red_ (−330 mV at pH 7) or glutathione (GSH) (−220 mV at pH 7) promoted CrATG3 monomerization, being the effect of GSH less pronounced due to its less electronegative redox potential (Fig. 1A). In contrast, oxidation with DTT_ox_, H_2_O_2_, or Cu^2+^ prevented CrATG3 monomerization and led to the detection of an oxidized form of monomeric CrATG3 (Fig. 1A).

Moreover, CrATG3 reduction and oxidation were fully reversible processes (Fig. 1B), strongly suggesting the involvement of a redox PTM in the regulation of this protein. Accordingly, increasing DTT_red_ concentrations or longer incubation with this reducer boosted CrATG3 monomerization (Supplemental Fig. S2, C and D) while raising H_2_O_2_ concentrations slightly increased the level of dimeric and oxidized monomeric CrATG3 (Supplemental Fig. S2E).

Next, we investigated whether ATG3 from other organisms is redox regulated. To this aim, we focused on ATG3 from the model yeast *S. cerevisiae* ATG3 (ScATG3) because this organism is evolutionarily distant from *Chlamydomonas* and the protein shares only 32% identity with CrATG3 (Supplemental Fig. S3, A and B). First, we performed a redox characterization of the recombinant ScATG3 (Supplemental Fig. S2F). As described for CrATG3, ScATG3 migrated as monomeric or dimeric forms in nonreducing gels depending on the redox conditions. We found that the presence of reducing agents, including βME (Supplemental Fig. S2G) or DTT_red_ (Fig. 1C), triggers ScATG3 monomerization, whereas the addition of H_2_O_2_ resulted in the detection of higher oligomerized forms of the protein (Fig. 1D). Both reduction and oxidation were found to be reversible, strongly suggesting that ScATG3 was also subjected to redox PTM (Fig. 1E).

**Figure 2.**
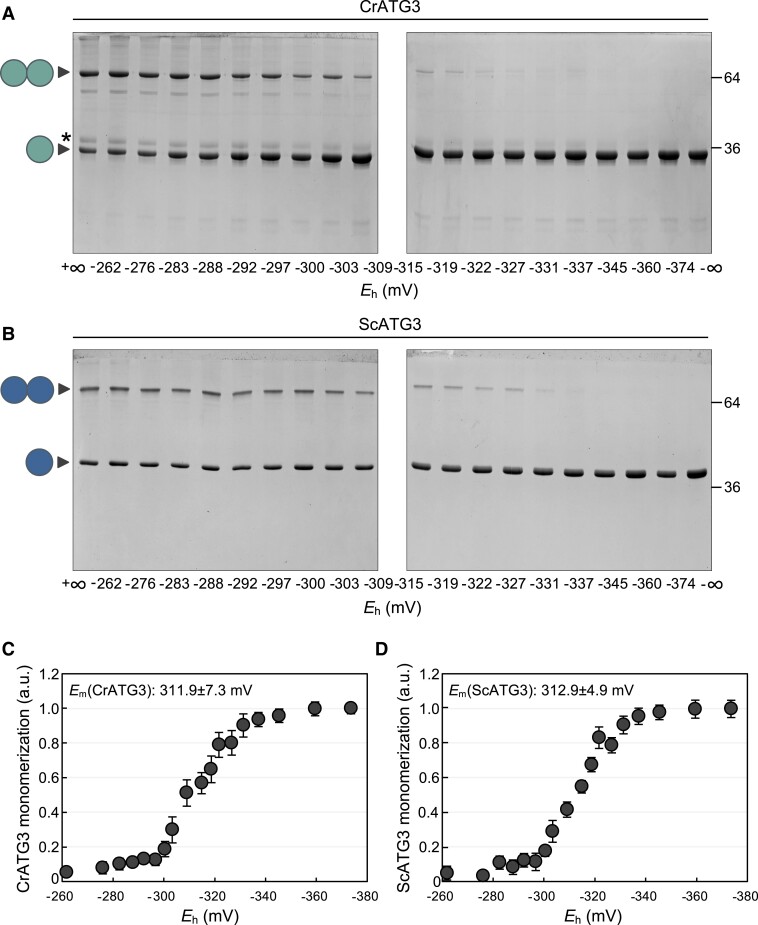
Monomerization of *Chlamydomonas* and *Saccharomyces* ATG3 proteins depends on redox potential. **A)** Redox titration of *Chlamydomonas* ATG3 (CrATG3) monomerization. The different isoforms (dimer, oxidized monomer, and reduced monomer) of *Chlamydomonas* ATG3 were analyzed after incubation at indicated *E*_h_ poised by 20 mM DTT in various dithiol/disulfide ratios. All samples were resolved by nonreducing SDS–PAGE gels and then visualized by Coomassie brilliant blue staining. The −∞ sample was considered 100% of CrATG3 reduced monomer and used as reference for quantification. **B)** Redox titration of *Saccharomyces* ATG3 (ScATG3) monomerization. Analysis of *Saccharomyces* ATG3 dimer/monomer ratio at the indicated *E*_h_ as described in **A)**. **C, D)** ATG3 monomerization as monitored in **A)** and **B)**, respectively, was quantified and interpolated by nonlinear regression of the data using the Nernst equation for 2 electrons exchanged (*n* = 2) and 1 redox component. The average midpoint redox potential (*E*_m,7.5_) of 3 independent experiments is shown in the figure as mean ± Sd. The symbol * shows oxidized and monomeric CrATG3. One ball and 2 balls correspond to reduced monomer and dimer, respectively, of CrATG3 or ScATG3.

The similar results obtained with 2 divergent ATG3 enzymes prompted us to further characterize their redox regulation. To investigate the link between ATG3 and the redox potential, we performed a complete DTT redox titration by varying the ambient redox potential (*E*_h_ at pH 7.5) between −262 and −374 mV. CrATG3 and ScATG3 proteins were incubated at each redox potential, and the ratio of ATG3 monomerization was determined by analyzing the electrophoretic mobility in nonreducing denaturing gels (Fig. 2). Our data revealed a strong association between the monomerization of both ATG3 proteins and the redox potential. Oxidized (dimer and oxidized monomer) and reduced (monomer) ATG3 forms were observed under less electronegative redox potential (from −262 to −322/−327 mV), although only the reduced monomeric form was detected at *E*_h_ values higher than −345 mV (Fig. 2, A and B). The experimental data of in-gel redox titrations gave good fits to the Nernst equation for the reduction of a single 2-electron component, with a midpoint redox potential (*E*_m_ at pH7.5) of −311.9 mV for CrATG3 (Fig. 2C) and −312.9 mV for ScATG3 (Fig. 2D), indicating the participation of a redox-regulated disulfide bond in the formation of ATG3 dimers in both proteins. In addition to this modification, CrATG3 showed a clear oxidation of the monomeric form under less electronegative redox potential (Fig. 2A) that was not evident for ScATG3 (Fig. 2B), suggesting that additional redox PTM may occur in vivo in CrATG3.

Thus, taking together, our results indicated that CrATG3 and ScATG3 are redox regulated. Moreover, the molecular mechanism of ATG3 redox regulation is likely conserved between yeasts and algae and involves a dithiol–disulfide exchange reaction with a very low midpoint redox potential.

### Trx efficiently reduces CrATG3

**Figure 3.**
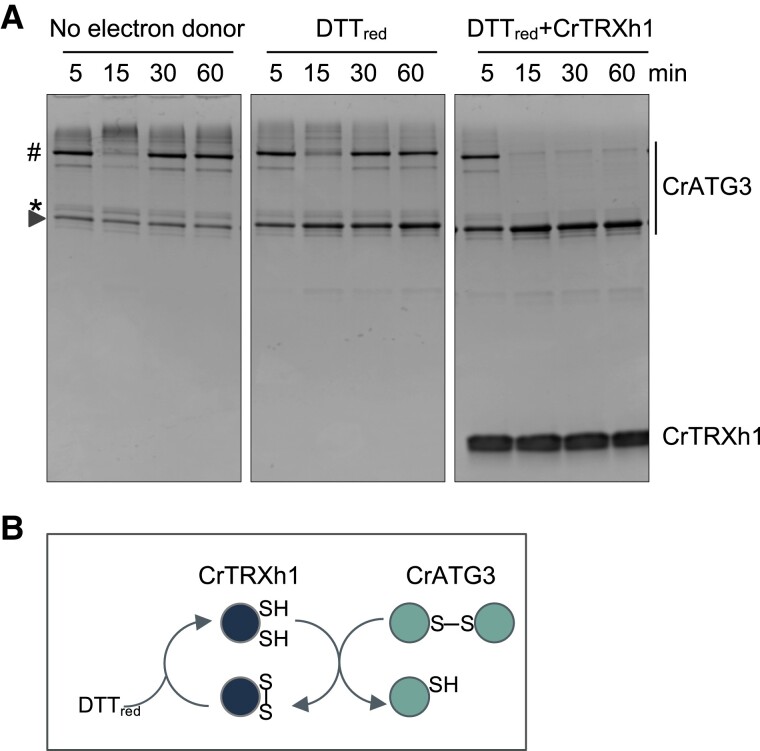
*Chlamydomonas* ATG3 is reduced by Trx. **A)** Monomerization of CrATG3 after incubation for the indicated times (5, 15, 30, and 60 min) in the presence of CrTRXh1 (5 *µ*M) (right panel). A low DTT_red_ concentration (0.25 mM) was used to keep CrTRXh1 at its reduced status. Samples with no reducer (left panel) or only with a low DTT_red_ concentration (middle panel) were used as controls. Proteins were subjected to nonreducing 15% SDS–PAGE gels and then visualized by Coomassie brilliant blue staining. The different CrATG3 isoforms and CrTRXh1 are indicated on the right. The reduced and monomeric CrATG3, the oxidized and monomeric CrATG3, and the dimeric CrATG3 are highlighted with an arrow, an asterisk (*), and a hash (#), respectively, on the left. **B)** Schematic representation of the proposed mechanism of monomerization of CrATG3 by the oxidoreductase CrTRXh1. Reduced CrTRXh1 (SH) is able to reduce the disulfide bond of dimeric CrATG3 resulting in reduced and monomerized CrATG3 (SH). DTT_red_ keeps CrTRXh1 in its reduced form. S–S represents a disulfide bond.

Trx are small oxidoreductases able to reduce disulfide bonds with very low redox potential in target proteins involved in a wide range of cellular processes such as the Calvin–Benson–Bassham (CBB) cycle or stress response (Buchanan and Balmer 2005; Pérez-Pérez et al. 2017). We have previously demonstrated that Trx plays a role in autophagy by regulating the ATG4 protease in yeasts and algae (Pérez-Pérez et al. 2014, 2016, 2021). Since our results indicated that ATG3 is subjected to redox modifications (Figs. 1 and 2; Supplemental Fig. S2), we investigated whether Trx may also regulate this essential autophagy protein. To this aim, we tested whether Trx is able to reduce the oxidized forms of CrATG3 by analyzing the time-course monomerization of CrATG3 using Trx or DTT_red_ as electron donors. Specifically, we incubated *Chlamydomonas* TRXh1 (CrTRXh1), the major cytosolic Trx (Lemaire and Miginiac-Maslow 2004), with CrATG3 for 5, 15, 30, and 60 min, and then analyzed the electrophoretic mobility of CrATG3 in nonreducing denaturing gels. Our data showed that CrTRXh1 efficiently reduces and monomerizes CrATG3 in the presence of 0.25 mM DTT_red_ (Fig. 3A), which is required to keep Trx reduced (Fig. 3B) (Buchanan and Balmer 2005). However, no effect on CrATG3 monomerization was detected in the absence of CrTRXh1 or when only low DTT_red_ was added (Fig. 3A). Moreover, the band corresponding to oxidized monomeric CrATG3 also decreased when samples were incubated with CrTRXh1 (Fig. 3A). Therefore, our findings indicated that Trx efficiently reduces CrATG3, suggesting a function of this oxidoreductase in the regulation of the redox state of ATG3.

### The catalytic Cys of ATG3 is involved in the redox regulation of ATG3

**Figure 4.**
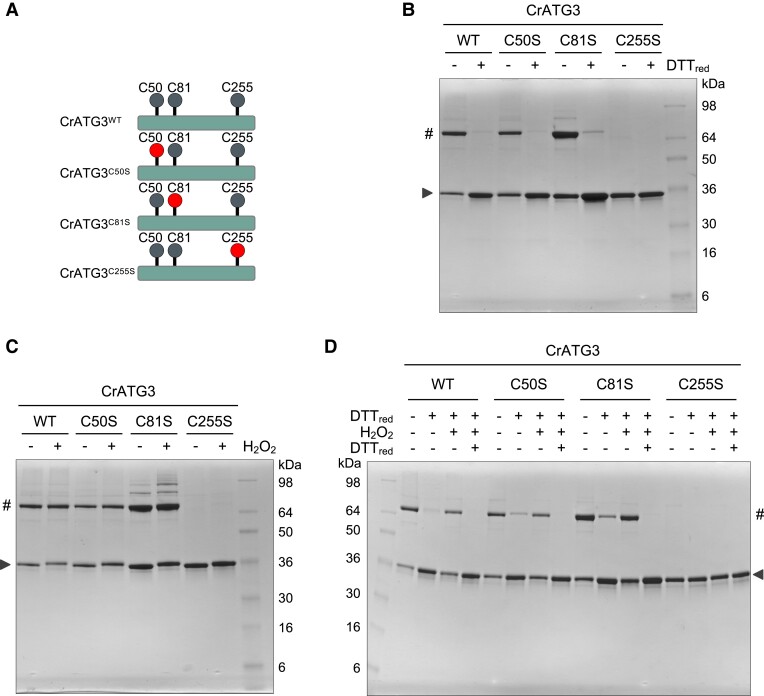
The catalytic Cys of *Chlamydomonas* ATG3 is subjected to redox regulation. **A)** Schematic representation of WT and Cys-to-Ser mutant versions of CrATG3: CrATG3^WT^, CrATG3^C50S^, CrATG3^C81S^, and CrATG3^C255S^. All Cys residues present in each CrATG3 version are depicted as gray balls, and the Cys-to-Ser mutation is highlighted as a red ball. **B)** Electrophoretic mobility of CrATG3^WT^ (WT), CrATG3^C50S^ (C50S), CrATG3^C81S^ (C81S), and CrATG3^C255S^ (C255S) in the absence (−) or presence (+) of 2.5 mM DTT_red_ for 30 min and then resolved by Coomassie brilliant blue-stained nonreducing SDS–PAGE gels. **C)** All versions of CrATG3 untreated (−) or treated (+) with 0.5 mM H_2_O_2_ for 20 min. **D)** Each CrATG3 version was sequentially incubated (+) with 0.5 mM DTT for 30 min, then with 2.5 mM H_2_O_2_ for 20 min, and newly with 10 mM DTT for 45 min. All incubation assays were performed at 25 °C using 2.5 *µ*g of each purified *Chlamydomonas* ATG3 version. In all experiments, untreated sample (−) was used as control. The arrow indicates monomeric CrATG3, and # symbol indicates CrATG3 dimer. The molecular mass marker (kDa) is shown.

The amino acid sequence of CrATG3 contains 3 Cys residues: the catalytic Cys (Cys255) and 2 N-terminal Cys (Cys50 and Cys81) (Fig. 4A; Supplemental Fig. S4; Mallén-Ponce et al. 2023). In order to identify the redox-sensitive Cys(s) of CrATG3, we analyzed the electrophoretic mobility of wild-type (WT) and Cys-to-Ser mutant versions of CrATG3 under different redox conditions in nonreducing denaturing gels (Fig. 4). Our data showed that mutation of catalytic Cys255 fully prevented the formation of dimeric CrATG3 regardless of the redox conditions (Fig. 4, B and C). On the contrary, the CrATG3^C50S^ and CrATG3^C81S^ mutants displayed the same behavior as CrATG3^WT^ when treated with DTT_red_ or H_2_O_2_, indicating that these 2 Cys do not participate in CrATG3 dimerization (Fig. 4, B and C). Moreover, as shown for CrATG3^WT^ (Fig. 1B), both reduction and oxidation of CrATG3^C50S^ and CrATG3^C81S^ are fully reversible when proteins are sequentially treated with 0.5 mM DTT_red_, 2.5 mM H_2_O_2_, and then 10 mM DTT_red_ (Fig. 4D). We also analyzed whether Cys255 is involved in the oxidation of monomeric CrATG3 by performing a DTT redox titration of this mutant form. Our results indicated that CrATG3^C255S^ is monomeric independently of the redox potential, and, interestingly, the oxidized monomeric CrATG3 form was not detected even under the lowest electronegative redox potential (Supplemental Fig. S5).

In close agreement with our experimental results, modeling of CrATG3 and ScATG3 proteins predicted that the catalytic Cys is the only highly conserved Cys in these proteins (Supplemental Fig. S4, A to D; Mallén-Ponce et al. 2023). Therefore, we also investigated the role of the catalytic Cys from ScATG3 (Cys234) in the redox regulation of this protein by analyzing the electrophoretic mobility of the Cys-to-Ser catalytic mutant ScATG3^C234S^ (Supplemental Fig. S2H). Our results showed that mutation of Cys234 precluded the dimerization of ScATG3 (Supplemental Fig. S2H). Taken together, our data strongly suggested that the catalytic Cys of ATG3 is subjected to redox regulation in algae and yeasts.

### ATG3 must be reduced to perform ATG8 lipidation

**Figure 5.**
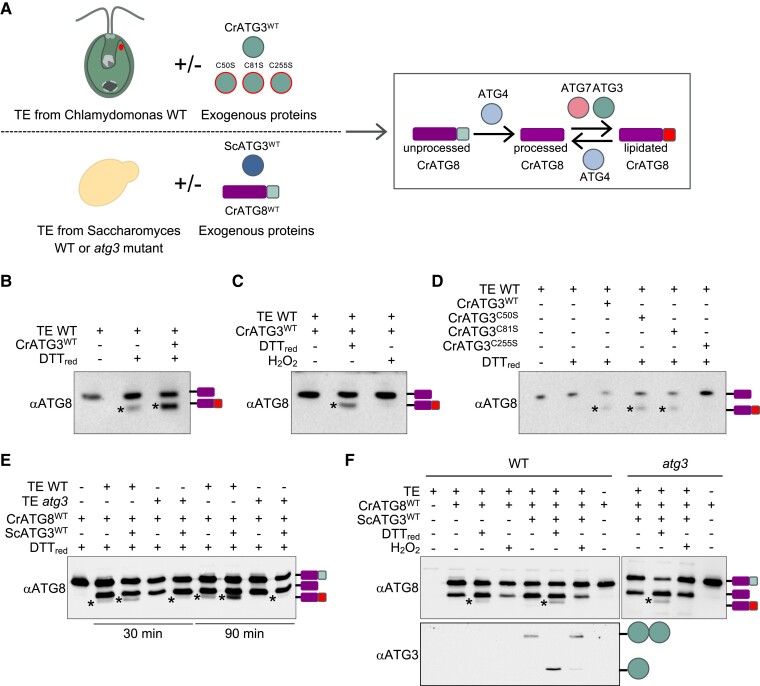
Reduced ATG3 mediates ATG8 lipidation in cell-free assays in *Chlamydomonas* and *Saccharomyces*. **A)** Schematic representation of the workflow of the cell-free CrATG8 lipidation assay using the recombinant E2-activating enzyme ATG3 (CrATG3^WT^, CrATG3^C50S^, CrATG3^C81S^, CrATG3^C255S^, or ScATG3^WT^), recombinant CrATG8 (CrATG8^WT^), and total extracts (TEs) from *Chlamydomonas* WT or *Saccharomyces* WT and *atg3* mutant. **B)** TEs from *Chlamydomonas* WT were untreated (−) or treated (+) with DTT_red_ in the absence (−) or presence (+) of recombinant CrATG3^WT^ for 60 min at 25 °C. **C)** TEs from *Chlamydomonas* WT were incubated with no agent (−), a reducer (DTT_red_), or an oxidant (H_2_O_2_) in the presence (+) of recombinant CrATG3^WT^ for 60 min at 25 °C. **D)** TEs from *Chlamydomonas* WT were untreated (−) or treated (+) with DTT_red_ in the presence (+) of recombinant CrATG3^WT^, CrATG3^C50S^, CrATG3^C81S^, or CrATG3^C255S^ for 60 min at 25 °C. **E)** TEs from *Saccharomyces* WT or *atg3* mutant strains were incubated in a DTT_red_-containing buffer in the absence (−) or presence (+) of recombinant His-tagged ScATG3 (ScATG3^WT^) for 30 or 90 min. **F)** TEs from *Saccharomyces* WT or *atg3* mutant strains were untreated (−) or treated (+) with DTT_red_ or H_2_O_2_ in the absence (−) or presence (+) of recombinant ScATG3^WT^ for 60 min at 25 °C. After the lipidation assay, proteins were resolved by 12% or 15% SDS–PAGE gel and finally analyzed by western blot with *Chlamydomonas* anti-ATG3 (αATG3) or anti-ATG8 (αATG8) antibodies. Lipidated ATG8 (ATG8-PE) is shown with an asterisk on the gel. The different ATG8 isoforms (unprocessed, processed, and lipidated ATG8) are depicted on the right.

ATG3 is an E2-activating enzyme that catalyzes the conjugation of ATG8 from ATG7 to the headgroup of PE (Ichimura et al. 2000) (Supplemental Fig. S1). To study the implications of CrATG3 redox regulation in the ATG8 conjugation process, we first set up a cell-free lipidation assay using total extract from *Chlamydomonas* and recombinant CrATG3 protein (Fig. 5A). This assay is based on a previous one performed to monitor ATG4 activity in total extracts from *Chlamydomonas* (Pérez-Pérez et al. 2010, 2016; Crespo and Pérez-Pérez 2022) and *Arabidopsis* (Laureano-Marín et al. 2020). ATG8 lipidation can be easily monitored in vivo in *Chlamydomonas* cells subjected to autophagy-activating conditions since processed and free ATG8 can be clearly distinguished from the PE-conjugated ATG8 form (Pérez-Pérez et al. 2010; Crespo and Pérez-Pérez 2022). Briefly, the cell-free lipidation assay included the following steps: (i) incubation of *Chlamydomonas* total extract with recombinant CrATG3, (ii) protein electrophoresis under nonreducing denaturing conditions, and (iii) immunoblot analysis with anti-CrATG8 antibodies. First, we analyzed CrATG8 lipidation from total extracts in the absence or presence of exogenous recombinant CrATG3^WT^. We found that incubation of *Chlamydomonas* total extracts with DTT_red_ promotes CrATG8-PE formation and, notably, the addition of exogenous CrATG3^WT^ further increased the level of lipidated CrATG8 (Fig. 5B; Supplemental Fig. S6A). Next, we monitored CrATG8 lipidation in total extracts incubated with CrATG3^WT^ under reducing (DTT_red_) or oxidizing (H_2_O_2_) conditions. While the addition of DTT_red_ triggered CrATG8-PE formation, no lipidation was detected under nonreducing or oxidizing conditions (Fig. 5C). To study the role of each Cys from CrATG3 in CrATG8 lipidation, we performed the cell-free assay with the different forms of CrATG3 (CrATG3^WT^, CrATG3^C50S^, CrATG3^C81S^, and CrATG3^C255S^) using DTT_red_ as electron donor. We found that the addition of CrATG3^WT^, CrATG3^C50S^, or CrATG3^C81S^ increases the conjugation of CrATG8 to PE, whereas the catalytic Cys mutant CrATG3^C255S^ had no effect on CrATG8 lipidation (Fig. 5D). These results indicated that the catalytic Cys is the only redox-sensitive Cys in CrATG3.

Next, we investigated whether the redox regulation of yeast ATG3 might have a role in ATG8 lipidation using a similar cell-free assay with yeast total extracts and recombinant ScATG3. In this assay, recombinant His-tagged CrATG8 protein was used as the substrate, and its lipidation state was monitored by western blot with anti-CrATG8 antibodies (Fig. 5A). Given that anti-CrATG8 from *Chlamydomonas* poorly recognizes yeast ATG8 (Pérez-Pérez et al. 2010), no crosstalk signal was detected with endogenous yeast ATG8. To validate our assay, we incubated total extracts from WT and *atg3* yeast strains with recombinant CrATG8 under reducing conditions (DTT_red_) in the absence or presence of recombinant ScATG3. As previously reported (Pérez-Pérez et al. 2010), recombinant CrATG8 was efficiently processed by endogenous ATG4 present in yeast total extracts (Fig. 5E). In this assay, an additional band of higher mobility was detected in total extracts from WT yeast cells. We confirmed this band corresponds to lipidated CrATG8 because it was not detected with total extracts from *atg3* mutant cells, which are unable to lipidate ATG8 (Tsukada and Ohsumi 1993; Ichimura et al. 2000). Our results indicated that endogenous yeast ATG3 lipidates CrATG8, and this lipidation was enhanced when exogenous ScATG3 was added to the reaction mix (Fig. 5E; Supplemental Fig. S6B). Moreover, the addition of exogenous ScATG3 allowed the lipidation of CrATG8 when total extract from *atg3* cells was used (Fig. 5E). A time-course assay indicated that CrATG8 lipidation increases over time and is potentiated by ScATG3 addition (Fig. 5E). Once validated, the cell-free assay allowed us to study the effect of the redox regulation of yeast ATG3 in CrATG8 lipidation. In close agreement with the results obtained with CrATG3 (Fig. 5C), CrATG8-PE was detected under reducing conditions (DTT_red_) but not upon nonreducing (no treatment) or oxidizing (H_2_O_2_) conditions in total extracts from WT cells (Fig. 5F). As expected, CrATG8-PE was not observed in the *atg3* mutant unless ScATG3^WT^ was included in the assay (Fig. 5E). Moreover, in close agreement with our in vitro data (Fig. 1), we detected exogenous ScATG3 in this assay as a monomer in the presence of DTT_red_ but not with H_2_O_2_ (Fig. 5E). Taken together, our findings strongly suggested that the ATG3-mediated lipidation of ATG8 is regulated by redox signals in algae and yeasts.

### ATG3 protein abundance increases upon chloroplast damage

**Figure 6.**
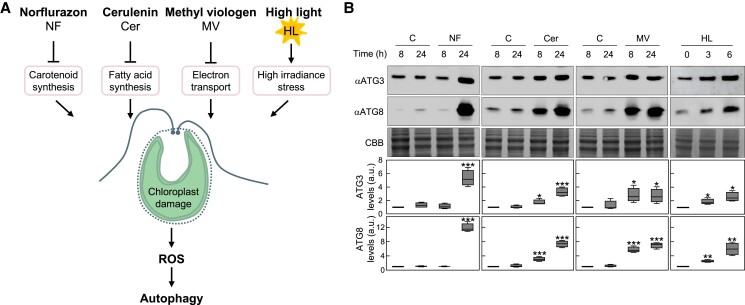
Autophagy-activating conditions lead to a significant increase of ATG3 abundance in *Chlamydomonas*. **A)** Schematic representation showing the effect of NF, cerulenin (Cer), or MV or HL in *Chlamydomonas* cells. All these treatments result in ROS accumulation that subsequently triggers the activation of autophagy (Pérez-Pérez, Couso, and Crespo 2012; Heredia-Martínez et al. 2018). **B)** Immunoblot analysis of *Chlamydomonas* ATG3 (upper panel) and ATG8 (middle panel) under different ROS-linked stress conditions such as: absence of carotenoids by treatment with 20 *µ*M NF (first column); fatty acid synthesis inhibition by treatment with 10 *µ*M cerulenin (second column); mitochondrial and chloroplast electron transport blocking by treatment with 1 *µ*M MV (third column); and HL stress (750 *µ*E/m^2^) (fourth column). Untreated cells **C)** were used as control. Coomassie brilliant blue-stained (CBB) gels were used as a protein loading control. Quantification of ATG3 levels (upper box plot) and ATG8 levels (lower box plot) from at least 4 biological replicates is shown. Boxplot elements: enter line, median; box limits, upper and lower quartiles; whiskers, min to max. Asterisks represent significant differences according to 2-tailed Student's *t*-test: ***P < 0.001; **P < 0.01; *P < 0.05; and not significant ≥0.05. The statistical analysis is detailed in Supplemental Table S2.

Our findings about the redox regulation of ATG3 in vitro and in cell-free extracts led us to investigate whether this protein is subjected to redox control in vivo under ROS-linked autophagy-activating conditions. To this aim, we focused on *Chlamydomonas* as we have previously demonstrated a clear regulation of autophagy by redox signals in this model organism. We have shown that chloroplast stress leads to enhanced ROS production and results in a strong induction of autophagy in *Chlamydomonas* (Fig. 6A). The addition of different drugs to *Chlamydomonas* cells, including norflurazon (NF), an herbicide that blocks carotenoid biosynthesis (Sandmann and Albrecht 1990); cerulenin, a fatty acid synthase enzyme inhibitor that prevents de novo fatty acid synthesis in the chloroplast (Moche et al. 1999); or MV, an electron acceptor that interrupts chloroplast and mitochondrial electron transport (Badger et al. 1980), notably increases ATG8 abundance and ATG8 lipidation in *Chlamydomonas* (Pérez-Pérez, Couso, and Crespo 2012; Pérez-Pérez et al. 2016; Heredia-Martínez et al. 2018; Fig. 6B). Similarly, a physiological chloroplast stress such as HL irradiance also activates autophagy in *Chlamydomonas* (Pérez-Pérez, Couso, and Crespo 2012; Fig. 6B). To investigate the in vivo redox regulation of CrATG3, we generated an antibody against this protein (see Materials and methods) that allowed us to monitor the abundance and redox state of CrATG3 in *Chlamydomonas* cells under different stress conditions. First, we checked that this antibody recognizes a 36-kDa band in total extracts corresponding to ATG3 protein (Supplemental Fig. S7). Then, using standard reducing gels and western blot analysis, we found that CrATG3 abundance increased in NF-treated cells (Fig. 6B), in close agreement with the upregulation of CrATG8 and autophagy detected under this stress (Fig. 6B) (Pérez-Pérez, Couso, and Crespo 2012). The level of CrATG3 also increased in response to other autophagy-activating conditions, such as treatment with cerulenin, MV, or HL stress (Fig. 6B). These findings indicated that CrATG3 protein abundance increases under ROS-linked stress in *Chlamydomonas* cells.

### ATG3 is reduced to ensure ATG8 lipidation under autophagy-activating conditions

**Figure 7.**
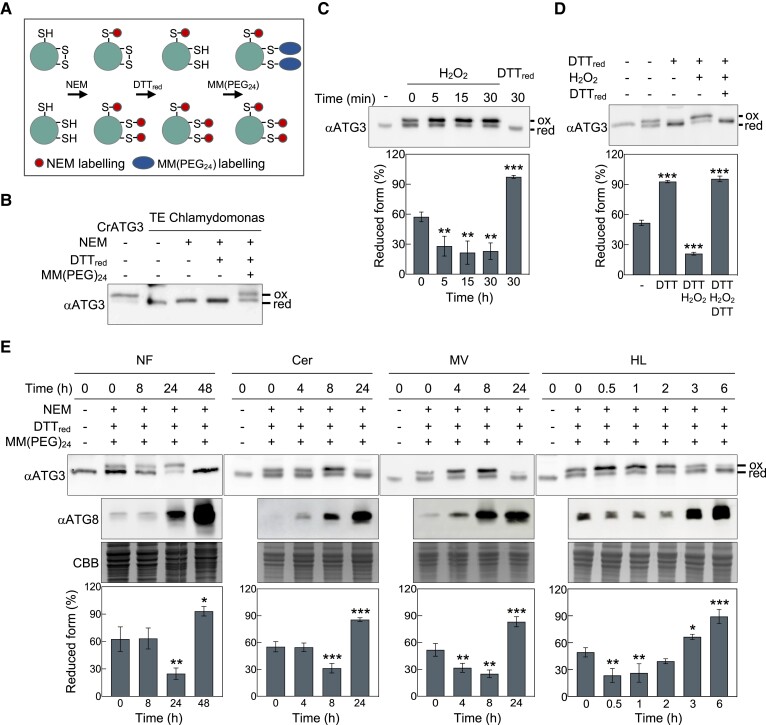
ROS-linked autophagy-activating conditions result in dynamic changes of ATG3 redox state in *Chlamydomona*s. A) Schematic representation of the in vivo redox alkylating assay. Briefly: (i) Sulfhydryl groups (SH) are blocked with NEM (red ball). (ii) Disulfide bonds (S–S) are reduced by DTT_red_. (iii) New SH groups are labeled with MM(PEG_24_) (blue ball). **B)** In vivo redox state of ATG3 in *Chlamydomonas*. Proteins were extracted from cells growing exponentially in TAP medium. Then, samples were untreated (−) or treated (+) sequentially with the alkylating (NEM), reducing (DTT_red_), and labeling-alkylating (MM(PEG)_24_) agents. Finally, *Chlamydomonas* ATG3 was visualized by western blot with anti-CrATG3. His-tagged recombinant CrATG3 (first lane) was used as molecular weight control. **C)** Total extracts (TEs) from *Chlamydomonas* WT were treated with an oxidant (1 mM H_2_O_2_) or a reducer (10 mM DTT_red_) for the indicated time on ice. After, samples were sequentially treated with the alkylating (NEM), reducing (DTT_red_), and labeling-alkylating (MM(PEG)_24_) agents and subjected to western blot with anti-CrATG3. **D)***Chlamydomonas* TEs were sequentially treated with 1 mM DTT_red_ (15 min), 10 mM H_2_O_2_ (15 min), and then 50 mM DTT_red_ (15 min), on ice, and finally processed as described in **C)**. **E)** In vivo redox state of ATG3 protein in cells subjected to NF, cerulenin (Cer), MV or HL stress. Proteins were obtained from cells before (0 h) and after treatment (at the indicated time) and labeled with the alkylating agent MM(PEG)_24_ as described in **A)**. Immunoblotting with anti-CrATG3 (upper panel) and anti-CrATG8 (middle panel). Coomassie brilliant blue-stained gels (CBB) were used as protein loading control (lower panel). ox, oxidized form; red, reduced form. Quantification of reduced ATG3 (%) from at least 3 independent experiments is shown. Error bars correspond to SD from 3 samples. Asterisks represent significant differences according to 1-way ANOVA and Bonferroni's test: ***P < 0.001; **P < 0.01; *P < 0.05; and not significant ≥0.05. The statistical analyses described apply to all statistical analyses in this figure, and they are detailed in Supplemental Table S2.

We examined the redox state of CrATG3 in *Chlamydomonas* cells upon autophagy activation using an in vivo alkylating assay with the blocking agents N-ethylmaleimide (NEM) and MM(PEG)_24_. NEM binds to free sulfhydryl groups and causes a negligible increase in the molecular weight of the protein (Yano 2003). The Cys labeling by NEM occurs only in reduced Cys, while MM(PEG)_24_ binds to originally oxidized Cys that were previously reduced by DTT_red_ in our assay (Fig. 7A) (Roberts et al. 2002; Yano 2003). MM(PEG)_24_ treatment caused an increase in the size of the protein (2.4 kDa) that can be detected by a shift in the mobility of the labeled protein (Fig. 7A). Using this in vivo alkylating approach, we were able to detect oxidized and reduced forms of CrATG3 in total extracts from *Chlamydomonas* (Fig. 7B). Next, we demonstrated that the shift in the in vivo redox state of CrATG3 is due to the presence of ROS. To this aim, we performed a cell-free assay incubating *Chlamydomonas* total extracts with H_2_O_2_ at different times. Our results indicated a pronounced increase of CrATG3 oxidation within 5 min of treatment (Fig. 7C). However, the addition of DTT_red_ resulted in the complete reduction of the protein (Fig. 7C). Moreover, the sequential incubation of *Chlamydomonas* total extracts first with DTT_red_, then with H_2_O_2_, and finally with DTT_red_ revealed that both reduction and oxidation of CrATG3 are fully reversible (Fig. 7D). Similar results were obtained with the opposite treatment (H_2_O_2_, DTT_red_, and then H_2_O_2_) (Supplemental Fig. S8).

Our results indicated that similar amounts of oxidized and reduced CrATG3 were detected in cells under optimal physiological growth conditions (Fig. 7, B and C), indicating that this protein is partially oxidized in the absence of stress. We then investigated whether stress might influence the redox state of CrATG3 by applying different ROS-generating stress conditions to *Chlamydomonas* cells. The in vivo alkylating analysis revealed profound changes in the redox state of CrATG3 in response to NF treatment. Under normal physiological growth or before autophagy activation, around 60% of total CrATG3 was reduced (Fig. 7E). However, induction of autophagy by NF, which takes place around 24 h after treatment (Pérez-Pérez, Couso, and Crespo 2012), led first to a pronounced oxidation of CrATG3 and then to a complete reduction of the protein at the time of the highest CrATG8 activation (48 h) (Fig. 7E). These results prompted us to investigate whether the marked redox change of CrATG3 in NF-treated cells was specific to the absence of carotenoids or a general response to ROS-linked stress in *Chlamydomonas*. To this purpose, we examined the redox state of CrATG3 in cerulenin- or MV-treated cells, which generate ROS and activate autophagy in response to different signals (Pérez-Pérez, Couso, and Crespo 2012; Heredia-Martínez et al. 2018). In both cases, CrATG3 was mainly oxidized after 8 h of treatment and then totally reduced after 24 h, when the highest ATG8 activation was observed (Fig. 7E). To further connect the redox regulation of CrATG3 with physiological oxidative stress, we analyzed the in vivo redox state of CrATG3 in *Chlamydomonas* cells exposed to HL (750 *µ*E/m^2^) irradiance. In close agreement with our results with NF, cerulenin, and MV, CrATG3 oxidation increased when cells were challenged to HL stress and then reduced CrATG3 markedly increased at the time of ATG8 activation.

Taken together, the in vivo redox analysis of CrATG3 suggested that this protein must undergo full reduction in order to promote CrATG8 lipidation and autophagy progression in *Chlamydomonas*.

## Discussion

Genomic and functional studies have demonstrated that the central machinery of autophagy is conserved in plants and microalgae, but the underlying molecular mechanisms regulating this catabolic process are not fully understood in the green lineage. In this study, we have shown that ATG3, a key autophagy protein required for ATG8 lipidation, is regulated through dynamic reduction/oxidation reactions to ensure autophagy progression under ROS-linked stress in *Chlamydomonas*.

The maintenance of intracellular redox balance is critical for photosynthetic organisms since electron transport in the chloroplast generates ROS that can oxidize cellular components (Li et al. 2009). Photosynthetic cells have multiple layers of defense against oxidative stress, including antioxidant compounds and antioxidant enzymes (Noctor et al. 2018). Other mechanisms contributing to maintain redox homeostasis in eukaryotic photosynthetic cells involve degradation pathways such as autophagy (Pérez-Pérez, Lemaire, and Crespo 2012; Signorelli et al. 2019). In addition to the deleterious effect of ROS, these molecules also act as important signaling molecules (Apel and Hirt 2004; Zaffagnini et al. 2018). Indeed, many proteins can be regulated by the redox state of key Cys residues, including the formation of disulfide bonds or other redox PTMs (Buchanan 1991; Michelet et al. 2013; Pérez-Pérez et al. 2017; Wakao and Niyogi 2021).

In order to tackle the harmful effects of ROS, cells upregulate autophagy to eliminate impaired components, including oxidized proteins, or even entire organelles whose dysfunctional activity might further increase ROS levels (Pérez-Pérez, Lemaire, and Crespo 2012; Signorelli et al. 2019). ROS also act as secondary messengers to modulate the activity of autophagy-related proteins (Scherz-Shouval et al. 2007). Biochemical analyses performed in humans (Scherz-Shouval et al. 2007), yeasts (Pérez-Pérez et al. 2014), and *Chlamydomonas* (Pérez-Pérez et al. 2016) revealed that ATG4 is regulated by redox signals.

Besides ATG4, the formation of ATG8-PE conjugates also depends on ATG7 and ATG3, and a recent study indicated that these 2 enzymes are also subjected to redox regulation in mammals. In vitro and in vivo data showed that the oxidation of ATG7 and ATG3 mediates the inhibition of autophagy through the formation of a hetero-disulfide bond that prevents the interaction and lipidation of LC3 (Frudd et al. 2018). The stable covalent complexes between LC3, ATG7, and ATG3 decrease upon autophagy activation when LC3 is transferred to PE. Moreover, the catalytic Cys of these 2 enzymes are prone to oxidation and establish a disulfide bond between ATG7 and ATG3 (Frudd et al. 2018). In close agreement with this study, our results showed that ATG3 proteins from *Chlamydomonas* and yeast are direct targets of ROS and undergo reversible redox PTMs. Our in-depth biochemical analysis demonstrated that the presence of ROS, such as H_2_O_2_, triggers the direct oxidation of ATG3 not only in vitro but also in vivo (Figs. 1 to 5 and 7). These results also suggested that the catalytic Cys of ATG3 is highly sensitive to oxidation, which occurs under nonstress conditions and is enhanced under ROS-provoking stresses.

E2-activating ATG3 activity and both proteolytic and deconjugating ATG4 activities should be tightly regulated in order to form ATG8-PE adducts under stress, and Trx, a major cellular thiol-based reductase, might play an important role in this control. We have previously reported that Trx reduces and activates ATG4 in yeast (Pérez-Pérez et al. 2014) and *Chlamydomonas* (Pérez-Pérez et al. 2016). In the current study, we found that ATG3 requires electron donors to carry out ATG8 lipidation and established this enzyme as a new Trx target (Fig. 3). This result is in close agreement with our previous Trx-targeted proteomic analysis in *Chlamydomonas*, where ATG3 was identified as a putative target of cytosolic Trx (Pérez-Pérez et al. 2017). In-gel redox titration assays indicated that *Chlamydomonas* and yeast ATG3 enzymes display a very low midpoint redox potential (*E*_m_ −311/−312 mV) (Fig. 2), suggesting that these proteins might be regulated by Trx due to its high reducing power (Gelhaye et al. 2004; Yoshida et al. 2018). The ATG3 redox potential values were more negative than those reported for *Chlamydomonas* and yeast ATG4 proteins (*E*_m_ −278 mV and *E*_m_ −289 mV, respectively) (Pérez-Pérez et al. 2014, 2016). Thus, we hypothesized that ATG4 and ATG3 are coordinately regulated according to the intracellular redox potential. During basal metabolism, the electronegative intracellular redox potential keeps ATG3 and ATG4 partially reduced and active. The proteolytic ATG4 and conjugating ATG3 activities promote ATG8-PE adduct formation, whereas the delipidating activity of ATG4 prevents excessive ATG8-PE formation, resulting in a basal level of lipidated ATG8 and housekeeping autophagy. By contrast, stress triggers ROS production, turning the redox potential into more electropositive and thus leading to sequential inhibition by oxidation of ATG4 and then ATG3. It is worth mentioning that the processing of ATG8 by ATG4 occurs constitutively (Kirisako et al. 2000; Pérez-Pérez et al. 2010), indicating that the ATG4 delipidating activity must be the one highly regulated (Nakatogawa et al. 2012; Yu et al. 2012; Abreu et al. 2017). The redox regulation of these autophagy proteins was accompanied by an upregulation of ATG3 (Fig. 6) and ATG4 protein abundance (Pérez-Pérez et al. 2016) upon ROS-mediated activation of autophagy, suggesting that these proteins participate in the cellular response of *Chlamydomonas* to oxidative stress.

Our in vitro results indicated that ATG3 displays 2 reversible oxidized isoforms in the presence of ROS inducers, an oxidized monomer, and a homodimer (Fig. 1); however, we could not detect dimeric ATG3 in total extracts from *Chlamydomonas* using anti-CrATG3 antibodies. Failure to detect oxidized dimers in vivo has been reported for other redox-regulated proteins. For instance, the elongation factor EF-Tu from the cyanobacterium *Synechocystis* is reversibly inhibited by oxidation, forming an intermolecular disulfide bond under oxidizing conditions in vitro while no oxidized dimers are detected in vivo due to immediate reduction by Trx (Jimbo et al. 2018). Given that ATG3 transiently interacts with ATG7 and ATG8 to transfer ATG8 to PE (Taherbhoy et al. 2011; Kaiser et al. 2012; Mallén-Ponce et al. 2023) and ATG3 can also interact with other proteins such as GAPDH (Han et al. 2015), it is plausible that these dynamic interactions prevent the covalent binding between ATG3 molecules and the detection of ATG3 dimers in *Chlamydomonas*. Otherwise, we cannot rule out that anti-CrATG3 antibodies do not recognize ATG3 oligomeric forms.

Here, we investigated the sensitivity of the catalytic Cys255 of CrATG3 to H_2_O_2_ and the effects of its oxidation on ATG8-PE conjugation activity using a cell-free assay with *Chlamydomonas* total extracts and recombinant proteins. Our results confirmed that ATG3 activity can be monitored in *Chlamydomonas* whole-cell extracts using endogenous ATG8 and recombinant ATG3 protein (Fig. 5). This assay allowed us to analyze the redox regulation of ATG8 lipidation by ATG3. A similar assay was initially set up to study ATG4 protease activity in *Chlamydomonas* (Pérez-Pérez et al. 2010), yeasts (Pérez-Pérez et al. 2014), and plants (Laureano-Marín et al. 2020). Therefore, this assay could be exploited for analyzing the regulation of ATG3 activity from other organisms, such as yeasts (Fig. 5, E and F), without the limitation of reconstituting the whole ATG8 lipidation system in vitro but using the components present in whole-cell extracts.

Reversible redox modifications allow proteins to quickly adapt their activity to fluctuating cellular requirements (Choudhury et al. 2017; Wakao and Niyogi 2021). Indeed, our time-course in vivo alkylation shift assays revealed the dynamics of CrATG3 redox regulation under different ROS-generating stress conditions in *Chlamydomonas* (Fig. 7). Remarkably, we found that the proportion of reduced ATG3 changed in accordance with ROS abundance likely to ensure ATG8 lipidation and autophagy progression. We have previously reported that NF generates ROS and activates autophagy in *Chlamydomonas* after 24 h and further stimulates this catabolic process at longer treatments (Fig. 6) (Pérez-Pérez, Couso, and Crespo 2012). Our results indicated that NF leads first to the oxidation of ATG3 at the time of autophagy induction (Fig. 7E), which is in close agreement with the inactivation of ATG4 reported in NF-treated cells (Pérez-Pérez et al. 2016). Similar results were obtained when Chlamydomonas cells were treated with cerulenin or MV, or by shifting cells from normal to HL. ATG3 was transiently oxidized in all these conditions, reaching the oxidation peak at the time of autophagy activation (Fig. 7E) (Pérez-Pérez, Couso, and Crespo 2012; Heredia-Martínez et al. 2018). However, ATG3 was detected almost exclusively in its reduced and active form under maximal autophagy induction (Fig. 7E), likely to boost ATG8 lipidation and promote autophagy progression. The similar redox-state pattern of ATG3 detected in cells treated with NF, cerulenin, or MV, and HL-stressed cells strongly suggests that the function of this essential autophagy protein is regulated by the production of ROS under different stress conditions. In consonance with our results, alkylation shift assays performed in mammalian cells also revealed increased oxidation of ATG3 in response to H_2_O_2_, which attenuates LC3 lipidation (Frudd et al. 2018). In mammals, it has also been proposed that ATG3 and ATG7 should be reduced to perform their conjugase activity in order to bind LC3 to PE. Consequently, the ATG3–ATG7 heterodimer formation decreases upon autophagy activation (Frudd et al. 2018). Whether ATG7 is also subject to redox regulation remains to be explored.

Taken together, our results unravel the molecular mechanisms underlying the redox regulation of ATG3, a main component of the ATG8 lipidation system. The coordinated regulation of different proteins from the ATG8 lipidation system, such as ATG3 and ATG4, would connect redox signals and antioxidant systems with the process of autophagy, which functions as a main mechanism to eliminate ROS-damaged components in photosynthetic organisms (Pérez-Pérez, Couso, and Crespo 2012; Signorelli et al. 2019).

## Materials and methods

### Strains, media, and growth conditions

The *Chlamydomonas* (*C. reinhardtii*) strain used in this study was WT 4A + (CC-4051), which was obtained from the Chlamydomonas Culture Collection. *Chlamydomonas* cells were grown under continuous illumination (50 *µ*mol photon m^−2^ s^−1^) on an orbital shaker (100 rpm) at 25 °C in Tris-acetate phosphate (TAP, pH 7.1) medium as previously described (Harris 1989).

The *Saccharomyces* (*S. cerevisiae*) strains used in this study were WT (SEY6210) and *atg3* mutant (MT33-1A) and were kindly provided by Dr. Daniel Klionsky (Tsukada and Ohsumi 1993). Yeast cells were grown in rich medium (YPD; 1% [w/v] yeast extract [Difco, 10215203], 2% [w/v] peptone [Difco, 211705], and 2% glucose [w/v] [Sigma-Aldrich, G7021]) on an orbital shaker (200 rpm) at 30 °C.

### Gene cloning and protein purification

The DNA-coding sequences of the *ATG3* genes from *C. reinhardtii* and *S. cerevisiae* were synthesized and cloned into pBluescript SK (+) vector by the company GeneCust Europe (genecust.com). *ATG3*-coding regions were cloned into the pET28a (+) plasmid (Novagen, 69864-3) for the expression of the corresponding His-tagged protein. *Chlamydomonas ATG3* was cloned at *Nde*I and *BamH*I sites of pET28a (+), whereas *Saccharomyces ATG3* was cloned at *BamH*I and *Xho*I sites.

The different *Chlamydomonas ATG3* mutants (*ATG3^C50S^*, *ATG3^C81S^*, and *ATG3^C255S^*) and *Saccharomyces ATG3* mutant (*ATG3^C234S^*) were synthesized by GeneCust Europe and cloned into pBluescript SK (+) vector. All *ATG3* mutants were also cloned into the pET28a (+) plasmid at the same restriction sites as WT versions. All clones were used to transform the *Escherichia coli* BL21 (DE3) strain. Recombinant proteins were expressed by induction at exponential growth phase (OD_600nm_ ≍ 0.5) with 0.5 mM isopropyl-β-D-thiogalactopyranoside (Sigma, I6758) for 2.5 h at 37 °C. All ATG3 recombinant proteins (WT and Cys-to-Ser mutants) were purified by affinity chromatography on a His-Select Nickel Affinity Gel (Sigma, P6611) following the manufacturer's instructions. The WT ATG8 from *Chlamydomonas* was purified as formerly described (Pérez-Pérez et al. 2010). The TRXh1^WT^ protein from *Chlamydomonas* was purified as described (Goyer et al. 1999). The *Chlamydomonas ATG3* sequence (Cre02.g102350) was obtained from Phytozome (phytozome-next.jgi.doe.gov), whereas the *Saccharomyces ATG3* (YNR007C) sequence was obtained from Yeast Genome Database (yeastgenome.org). All recombinant proteins used in this study are listed in Supplemental Table S1.

### In vitro protein analysis

To investigate the electrophoretic mobility of ATG3 proteins, the typical reaction mixture included 3 *µ*g of ATG3 in 50 mM Tris–HCl (pH 7.5) in the absence or presence of reducing (DTT_red_ [Sigma-Aldrich; 43815] or GSH [Sigma-Aldrich; G4251]) or oxidizing (DTT_ox_ [Sigma-Aldrich; D3511], H_2_O_2_ [Sigma-Aldrich; H1009], or CuSO_4_ [Sigma-Aldrich; 451657]) at the indicated concentration and time. For the analysis of Trx activity, 5 *µ*g of *Chlamydomonas* TRXh1 and 0.5 mM DTT_red_ as electron donor were used at the indicated incubation time. The reaction mixtures were incubated at 25 °C for the indicated time and stopped by the addition of βME-free loading sample buffer followed by 5 min at 65 °C. Proteins were resolved on 12% or 15% SDS–PAGE gels, stained with Coomassie brilliant blue (Sigma-Aldrich, 27816) and visualized using a ChemiDoc Imaging System (Bio-Rad, 17001401). When required, protein signals were quantified using the ImageLab software (Bio-Rad), and a typically untreated sample was used as reference.

### Redox titration

Redox titration of ATG3 monomerization was performed by incubating the recombinant ATG3 protein from *Chlamydomonas* or *Saccharomyces* in 50 mM Tris–HCl (pH 7.5) at defined *E*_h_ values imposed by reduced (DTT_red_) and oxidized (DTT_ox_) DTT in different dithiol/disulfide ratios (with 20 mM as final concentration of total DTT [DTT_red_ + DTT_ox_]) as previously described (Pérez-Pérez et al. 2014, 2016). ATG3 samples were equilibrated in redox buffers for 30 min at 25 °C, and then the reaction was stopped by the addition of βME-free loading sample buffer followed by 5 min at 65 °C. Then, proteins were separated by 12% SDS–PAGE gels. Proteins were visualized by Coomassie blue staining, and the ATG3 monomer and dimer were quantified using the ImageLab software (Bio-Rad); the sample treated with 20 mM DTT_red_ and 0 mM DTT_ox_ (*E*_h_ = −∞) was used as reference and considered totally reduced. Titration data were fitted to the Nernst equation (*n* = 2, 1 component). The midpoint redox potential (*E*_m_) is reported as the mean ± Sd of 3 independent experiments. The redox potential values (*E*_h_) are expressed at pH 7.5.

### Protein preparation from *Chlamydomonas* cells

Chlamydomonas cells from liquid cultures were collected by centrifugation (4,000 × *g* for 4 min), washed in lysis buffer (50 mM Tris–HCl, pH 7.5), and resuspended in a minimal volume of the same buffer. Cells were lysed by 2 cycles of slow freezing to −80 °C followed by thawing at room temperature. The soluble cell extract was separated from the insoluble fraction by centrifugation (15,000 × *g* for 20 min at 4 °C). Proteins were quantified with the Coomassie dye-binding method (BioRad, 500-0006) as described by the manufacturer.

### Immunoblot analysis

For immunoblot analyses, total protein extracts (10 to 20 *µ*g) were subjected to 12% or 15% SDS–PAGE and then transferred to 0.45 *µ*m nitrocellulose membranes (GE Healthcare, 10600003). The anti-CrATG3 polyclonal antibody was produced by injecting the recombinant WT CrATG3 protein into a rabbit using standard immunization protocols at the Animal Resource facility from the University of Sevilla. Anti-CrATG3 was diluted 1:15,000; anti-CrATG8 (Pérez-Pérez et al. 2010), anti-CrATG4 (Pérez-Pérez et al. 2016), and secondary rabbit antibodies were diluted 1:3,000, 1:5,000, and 1:10,000, respectively, in phosphate-buffered saline (PBS) containing 0.1% Tween 20 (AppliChem, A4974) and 5% milk powder (AppliChem, A0830). The Luminata Crescendo Millipore immunoblotting detection system (Millipore, WBLUR0500) was used to detect proteins with horseradish peroxidase-conjugated antirabbit secondary antibodies (Sigma, A6154).

### Autophagy assay

In this study, autophagy was triggered by the following stimulus: treatment with 20 *µ*M NF (Sigma-Aldrich, 34364) for 0, 8, 24, and 48 h; treatment with 10 *µ*M cerulenin (Sigma-Aldrich, C2389) for 0, 4, 8, and 24 h; treatment with 0.1 *µ*M MV (Sigma-Alrich, 85617) for 0, 4, 8, and 24 h; or exposition to HL conditions (750 *µ*mol photon m^−2^ s^−1^) for 0.5, 1, 2, 3, and 6 h. When required, cells growing exponentially (≍10^6^ cells/mL) were exposed to these autophagy-linked stress conditions, and samples were processed as indicated below and subjected to standard or redox western blot analyses with anti-CrATG3 antibodies. In the experiments of this study, autophagy induction was confirmed by a single readout, such as the detection of ATG8 modification (Pérez-Pérez et al. 2010; Klionsky et al. 2021a). Although ATG8 lipidation is necessary but not sufficient to induce autophagy, we have previously demonstrated the activation of autophagy under the different stress conditions tested in this study (Pérez-Pérez, Couso, and Crespo 2012; Pérez-Pérez et al. 2016; Heredia-Martínez et al. 2018).

### Cell-free ATG8 lipidation assays in *Chlamydomonas* and *Saccharomyces*

*Chlamydomonas* total cell extracts prepared as described above were incubated at 25 °C in the absence or presence of reducing (DTT_red_) or oxidizing (H_2_O_2_) agents for the indicated concentration and time and stopped by the addition of βME-free loading sample buffer followed by 5 min at 65 °C. Then, proteins were resolved by 15% SDS–PAGE and analyzed by western blot with anti-ATG8 (Pérez-Pérez et al. 2010) as described above.

For ATG8 lipidation assays in yeasts, cells were collected by centrifugation (4,000 × *g* for 4 min), washed in lysis buffer 1 (50 mM Tris–HCl, pH 7.5), resuspended in lysis buffer 2 (50 mM Tris–HCl, pH 7.5; 1 mM Pefabloc [Sigma-Aldrich, 76307]; and 0.1% [v/v] Triton X-100 [Sigma-Aldrich, T9284]) and vortexed 10 times for 30 s each time in the presence of glass beads (Sigma-Aldrich, G8772). Total cell extract was separated by centrifugation (15,000 × *g* for 20 min at 4 °C). Proteins were incubated at 25 °C with recombinant *Chlamydomonas* His-tagged ATG8 in the absence or presence of DTT_red_/H_2_O_2_, and ATG8 lipidation was analyzed by western blot analysis with *Chlamydomonas* anti-ATG8 antibody (Pérez-Pérez et al. 2010) as described above.

### Redox western blots

Cys-alkylation assays were performed using NEM (Sigma-Aldrich, E1271) and Methyl-PEG-Maleimide Reagent (MM(PEG)_24_) (Thermo Fisher, 22713). NEM was added to *Chlamydomonas* cultures growing as indicated in each case to a final concentration of 10 mM. After 15 min of NEM incubation, cells were collected by centrifugation (4,000 × *g* for 4 min), washed in alkylating buffer (50 mM Tris–HCl, pH 7.5; 50 mM NaCl; and 10 mM NEM), and resuspended in a minimal volume of the same buffer. Cells were lysed by 2 cycles of slow freezing to −80 °C followed by thawing at room temperature. The soluble cell extract was separated from the insoluble fraction by centrifugation (15,000 × *g* for 20 min at 4 °C) and incubated with 10% (w/v) of TCA (Sigma-Aldrich, T6399) for 30 min on ice. Then, samples were centrifugated (15,000 × *g* for 20 min at 4 °C), and precipitates were washed twice with ice-cold acetone and resuspended in an SDS-containing buffer (50 mM Tris–HCl, pH 7.5; 50 mM NaCl; and 2% [w/v] SDS). Next, samples were incubated with 100 mM DTT_red_ for 60 min on ice. Finally, samples were again treated with TCA and ice-cold acetone, and precipitates were resuspended in a new buffer (50 mM Tris–HCl, pH 8.0; 50 mM NaCl; 2% [w/v] SDS, 7.5% [v/v] glycerol; and 0.01% bromophenol blue) in the absence or presence of 10 mM MM(PEG)_24_.

### Accession numbers

Sequence data from this article can be found in the NCBI (https://www.ncbi.nlm.nih.gov/pubmed) data library under accession numbers: CrATG3: *C. reinhardtii* (EDP07491); VcATG3: *Volvox carteri* (EFJ46364); CvATG3: *Chlorella variabilis* (EFN54110); OlATG3: *Ostreococcus lucimarinus* (ABO96836); Pt: *Phaeodactylum tricornutum* (EEC51122); At: *A. thaliana* (OAO94560); ZmATG3: *Zea mays* (ACJ72033); OsATG3: *Oryza sativa* (EEE54060.1); SpATG3: *Schizosaccharomyces pombe* (CAA17786); *S. cerevisiae* (KZV08635); DmATG3: *Drosophila melanogaster* (NP_649059); and *Homo sapiens* (AAH02830).

## Supplementary Material

kiad520_Supplementary_Data

## Supplemental data

The following materials are available in the online version of this article.

**Supplemental Figure S1.** The ATG8 and ATG12 ubiquitin-like systems in autophagy.

**Supplemental Figure S2.** His-tagged recombinant ATG3 proteins from *Chlamydomonas* (CrATG3) and *Saccharomyces* (ScATG3) purified in *E. coli*.

**Supplemental Figure S3.** Comparison of ATG3 enzymes from different organisms.

**Supplemental Figure S4.** Predicted structure of ATG3 enzyme from *Chlamydomonas* based on the structure of *Saccharomyces* ATG3 (PDB ID: 2DYT).

**Supplemental Figure S5.** Redox titration of *Chlamydomonas* catalytic Cys-to-Ser CrATG3 mutant protein monomerization.

**Supplemental Figure S6.** Reduced ATG3 mediates *Chlamydomonas* ATG8 lipidation in cell-free assays.

**Supplemental Figure S7.** CrATG3 antibodies recognize exogenous and endogenous CrATG3 protein.

**Supplemental Figure S8.** Reversibility of *Chlamydomonas* ATG3 redox state in cell-free total extracts.

**Supplemental Table S1.** Proteins used in this study.

**Supplemental Table S2.** Statistical analysis of main figures.

## Dive Curated Terms

The following phenotypic, genotypic, and functional terms are of significance to the work described in this paper:

atg3 Gramene: AT5G61500
atg3 Araport: AT5G61500
