# Supplementary material for: Redox-mediated activation of ATG3 promotes ATG8 lipidation and autophagy progression in Chlamydomonas reinhardtii
Source: Plant Physiol. 2023 Sep 29;194(1):359–75. doi: 10.1093/plphys/kiad520 (PMC10756753; doi:10.1093/plphys/kiad520)
Supplement: kiad520_Supplementary_Data [file kiad520_supplementary_data.pdf]

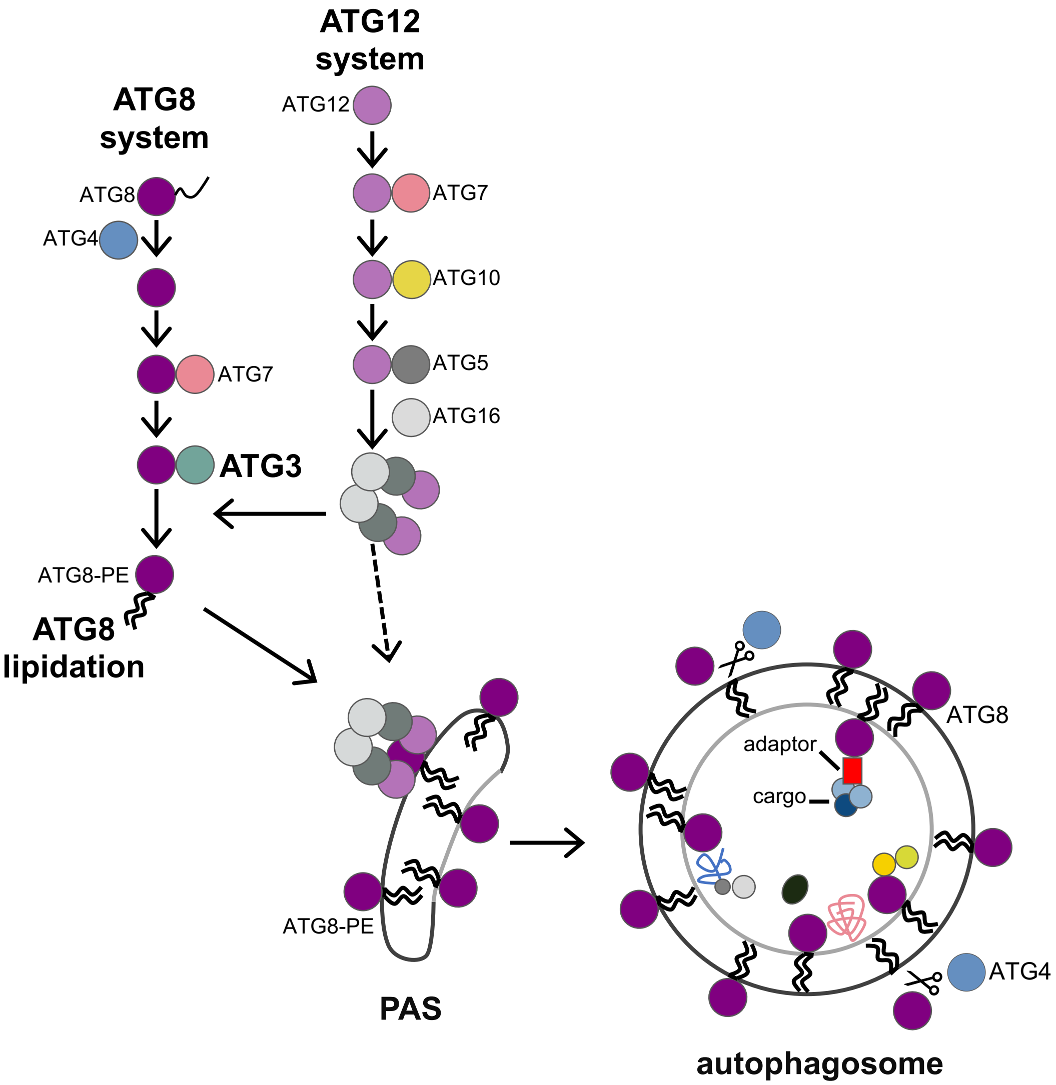

Supplemental Figure S1. The ATG8 and ATG12 ubiquitin-like systems in autophagy. After autophagy initiation, the components of ATG8 (ATG3, ATG4, ATG7 and ATG8 proteins) and ATG12 (ATG5, ATG7, ATG10, ATG12 and ATG16 proteins) systems are recruited to the preautophagosomal structure or PAS. All of these proteins act coordinately and sequentially to promote autophagosome biogenesis. The conjugation of ATG8 to the membrane phospholipid phosphatidylethanolamine (PE) is a key step in vesicle expansion and autophagosome formation. For ATG8 lipidation, first, nascent ATG8 is cleaved at a highly conserved Gly at its C-terminus by the Cys-protease ATG4. The C-terminal Gly is then activated by the E1 enzyme ATG7, with ATP consumption, and transferred to the E2 enzyme ATG3. Finally, ATG8 is bound to the headgroup of PE, and the ATG8-PE adduct is formed. The ATG5-ATG12-ATG16 protein complex acts as an E3 enzyme to stimulate the binding of ATG8 to PE. Besides processing ATG8 precursor, ATG4 presents a deconjugating or delipidating activity to cleave ATG8-PE and release free ATG8 from autophagosome membranes for recycling. The lipidation of ATG8 also plays an important role in cargo recognition since ATG8-PE interacts specifically with the intracellular material that will be engulfed into the autophagosome directly or through adaptors.

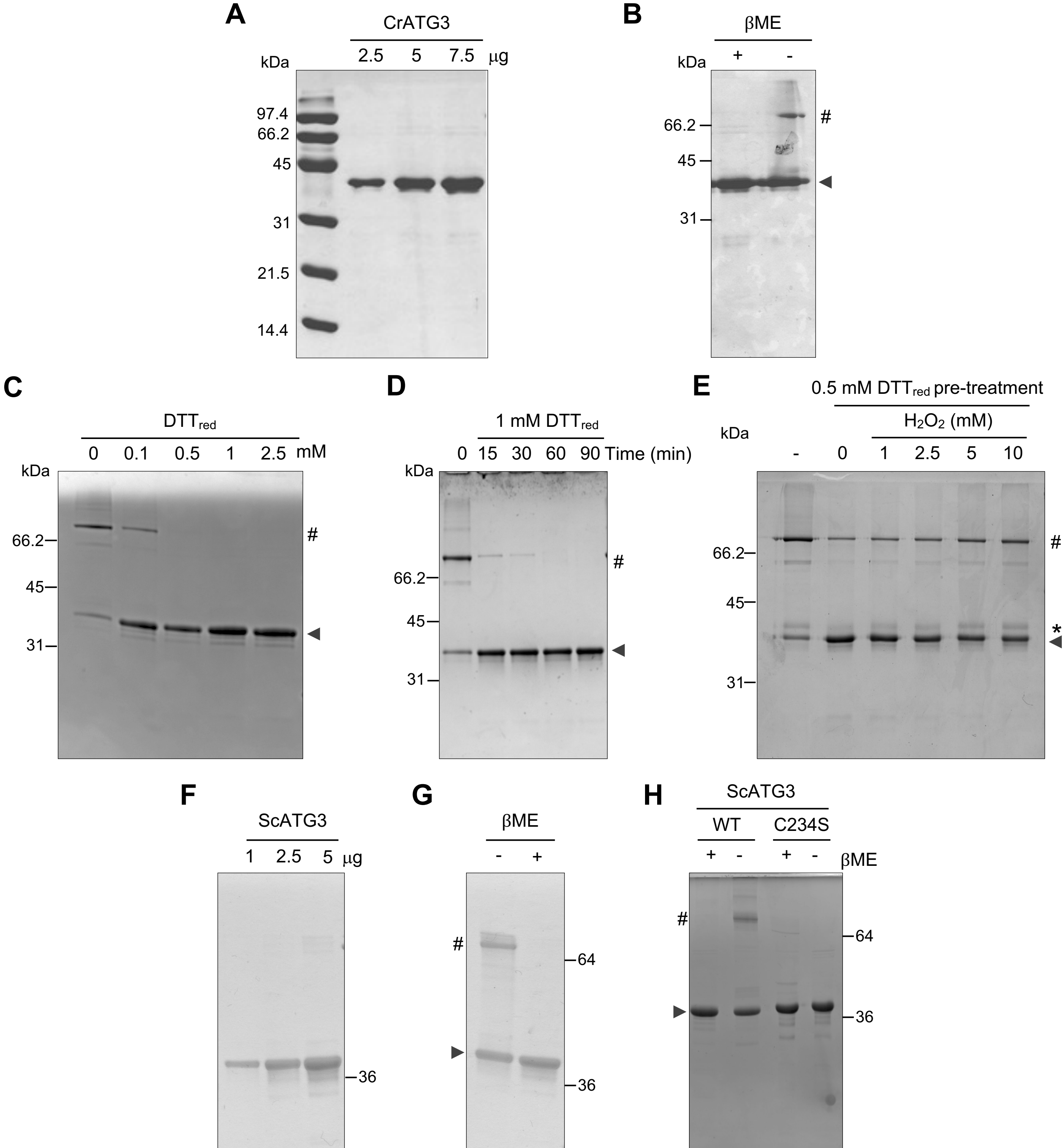

Supplemental Figure S2. His-tagged recombinant ATG3 proteins from Chlamydomonas (CrATG3) and Saccharomyces (ScATG3) purified in *E. coli*. A. Increasing concentrations (2.5, 5 and 7.5 μg) of CrATG3 in a β-mercaptoethanol (βME)-containing loading buffer subjected to SDS-PAGE gel. **B.** Electrophoretic mobility of CrATG3 in the absence (-) or presence (+) of the reducer βME in a non-reducing SDS-PAGE gel. **C.** CrATG3 treated with increasing concentrations of DTT_{red} (0, 0.5, 1 and 2.5 mM) for 30 min. **D.** CrATG3 treated with 1 mM DTT_{red} (0, 15, 30, 60 and 90 min). **E.** CrATG3 in the presence of increasing concentrations of H_2O_2 (0, 1, 2.5 and 5 mM) for 30 min after pretreatment with DTT_{red} (0.5 mM, 60 min). **F.** Increasing concentrations (1, 2.5 and 5 μg) of ScATG3 in a βME-containing loading buffer subjected to SDS-PAGE gel. **G.** Electrophoretic mobility of ScATG3 in the absence (-) or presence (+) of the reducer βME in a non-reducing SDS-PAGE gel. **H.** $ScATG3^{WT}$ (WT) and $ScATG3^{C234S}$ (C234S) in the absence (-) or presence (+) of βME in a non-reducing SDS-PAGE gel. The molecular mass marker (kDa) is shown. The different CrATG3 isoforms are indicated with an arrow (reduced monomeric ATG3), an asterisk (*) (oxidized monomeric ATG3) and a hash (#) (dimeric ATG3).

A

Organism	ATG3 Chlamydomonas (% Identity)	Amino acid number (Cys number)
Chlamydomonas reinhardtii	**100**	**306 (3)**
Volvox carteri	69.3	332 (3)
Chlorella variabilis	53.1	330 (3)
Ostreococcus lucimarinus	43.6	290 (3)
Phaeodactylum tricornutum	38.7	302 (4)
Arabidopsis thaliana	50.6	313 (3)
Zea mays	50.3	311 (3)
Oryza sativa	49.5	316 (3)
Schyzosaccharomyces pombe	34.2	275 (4)
Saccharomyces cerevisiae	**32**	**310 (4)**
Drosophila melanogaster	41.2	330 (5)
Homo sapiens	36.9	317 (8)

B

```
CrATG3          MSNLRHTLHTLFKQTVETVTPPLTKSQFEEKRVLTPDEFVAAGDYLVHACPTWSWEGGDP  60
ScATG3          ------MIRSTLSSWREYLTPITHKSTFLTTGQITPEEFVQAGDYLCHMFPTWKWNEESS  54
                       ::: :..  * :**   ** *  .  :**:*** ***** *  ***.*:  .

CrATG3          -KKRRTYFPPNKQFLVTRNVPCLKRATELEGYNPNSEFDVGGG-EGEDAWVATHSNPAAA  118
ScATG3          DISYRDFLPKNKQFLIIRKVPCDKRAEQCVEVEGPDVIMKGFAEDGDED-----------  103
                  . * ::* *****: *:*** *** :    :  . :  * . :*::

CrATG3          SGSAGKGEVPSIDGAGAGGSGGAGAAGGNKDDDIPDITD----LELNEADDEAAAPSGRP  174
ScATG3          -------DVLEYIGSET-EHVQSTPAGGTKDSSIDDIDELIQDMEIKEEDEN---DDTEE  152
                       :* .  *: :     :  ***.**..* ** :    :*::* *::    . .

CrATG3          YLRAEEPADNIMRTRTYDLYITYDQYYQVPRFWLVGHDESRKPLLPQQVMEDVSEEHARK  234
ScATG3          FNAKGGLAKDMAQERYYDLYIAYSTSYRVPKMYIVGFNSNGSPLSPEQMFEDISADYRTK  212
                :      *.:: : * *****:*.  *:**:::**.:..  .** *:*::**:* ::  *

CrATG3          TITVDPHPHLA-GLSAASIHPCRHADVMKKLVDNLLE-----------------------  270
ScATG3          TATIEKLPFYKNSVLSVSIHPCKHANVMKILLDKVRVVRQRRRKELQEEQELDGVGDWED  272
                * *::  *.   .: :.*****:**:*** *:*::

CrATG3          ----AGREFKVEQYLVLFLKFIASVVPTIQYDYTMSVGGE* 306
ScATG3          LQDDIDDSLRVDQYLIVFLKFITSVTPSIQHDYTMEGW--- 310
                     . .::*:***::*****:**.*:**:****.
```

Supplemental Figure S3. Comparison of ATG3 enzymes from different organisms. A. Table showing the identity (%) of ATG3 proteins from different species (*Chlamydomonas reinhardtii; Volvox carteri; Chlorella variabilis; Ostreococcus lucimarinus; Phaeodactylum tricornutum; Arabidopsis thaliana; Zea mays; Oryza sativa; Schyzosaccharomyces pombe; Saccharomyces cerevisiae; Drosophila melanogaster* and *Homo sapiens*). The identity was obtained by alignment of the two sequences using Pairwise Sequence Alignment EMBOSS Needle. The identity (%), the amino acid and cysteine residues numbers (in parentheses) are indicated. The two organisms used in this study (*Chlamydomonas reinhardtii* and *Saccharomyces cerevisiae*) are highlighted in bold. The accession numbers and the species abbreviations are indicated. CrATG3: *Chlamydomonas reinhardtii* (EDP07491); VcATG3: *Volvox carteri* (EFJ46364); CvATG3: *Chlorella variabilis* (EFN54110); OlATG3: *Ostreococcus lucimarinus* (ABO96836); Pt: *Phaeodactylum tricornutum* (EEC51122); At: *Arabidopsis thaliana* (OAO94560); ZmATG3: *Zea mays* (ACJ72033); OsATG3: *Oryza sativa* (EEE54060.1); SpATG3: *Schyzosaccharomyces pombe* (CAA17786); *Saccharomyces cerevisiae* (KZV08635); DmATG3: *Drosohpila melanogaster* (NP_649059); *Homo sapiens* (AAH02830). ATG3 sequences from all organisms were obtained from NCBI (https://www.ncbi.nlm.nih.gov/pubmed). **B.** Amino acid sequence alignment of CrATG3 and ScATG3. The proteins were aligned with the Clustal Omega software. All Cys residues are highlighted in red. The identical residues in both sequences are shown in bold.

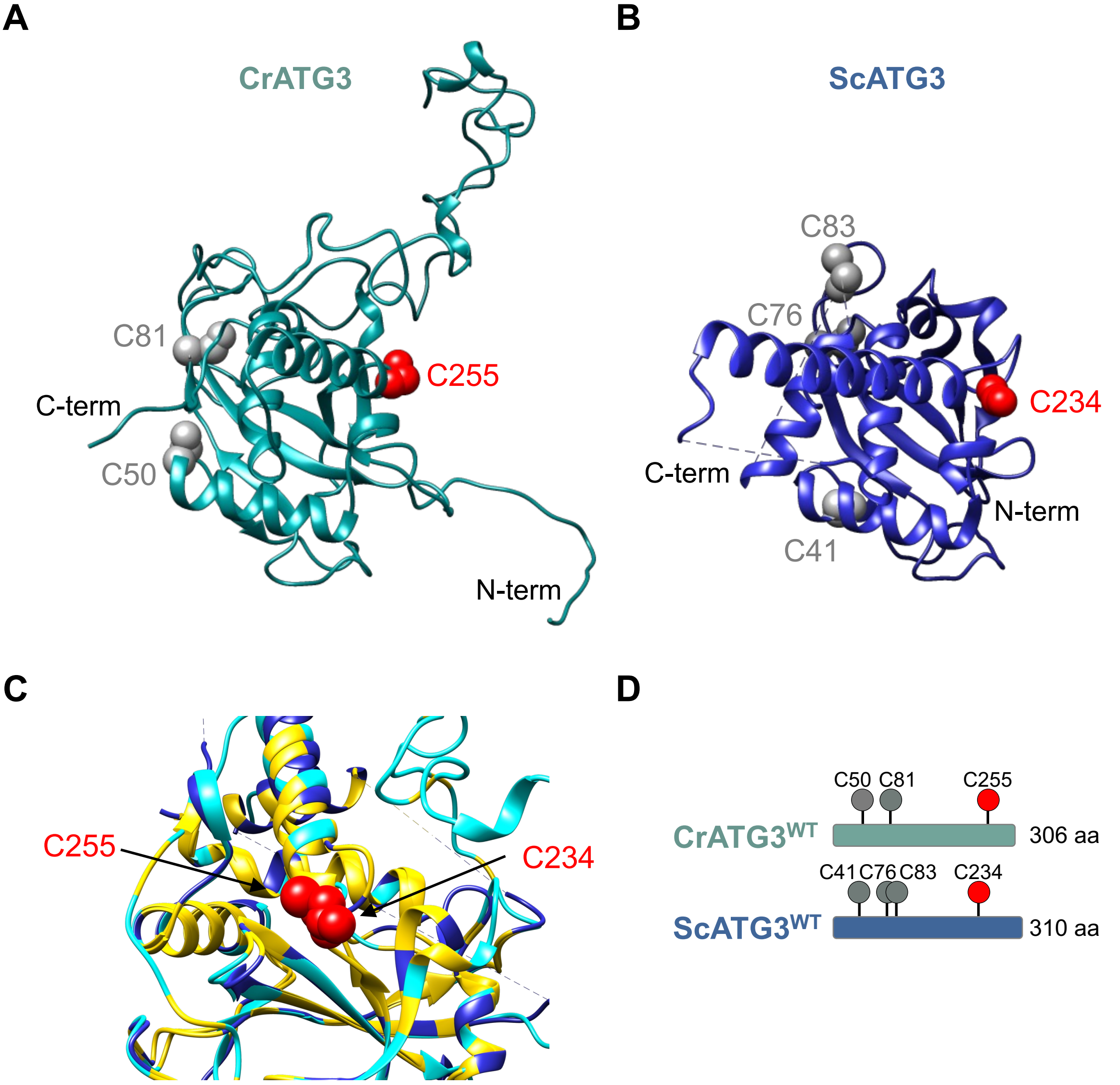

Supplemental Figure S4. Predicted structure of ATG3 enzyme from Chlamydomonas based on the structure of Saccharomyces ATG3 (PDB ID: 2DYT). A-B. Predicted structure of CrATG3 *(A)* and ScATG3 *(B)* showing all Cys of each protein and the N- and C-terminal regions. **C.** Enlarged overlapping of both predicted structures showing the catalytic Cys (Cys255 in CrATG3 and Cys234 in ScATG3) and the highly conserved area surrounding the catalytic Cys. CrATG3 is shown in turquoise, ScATG3 in blue, and the overlapping structure of both ATG3 is shown in yellow. **D.** Schematic representation of CrATG3 and ScATG3 showing the amino acid number. All Cys residues present in each ATG3 sequence are depicted: the highly conserved catalytic Cys (Cys255 in Chlamydomonas and Cys234 in Saccharomyces) is highlighted as a red ball, whereas the rest of Cys (Cys50 and Cys81 in Chlamydomonas; Cys71, Cys76 and Cys83 in Saccharomyces) are indicated as grey balls.

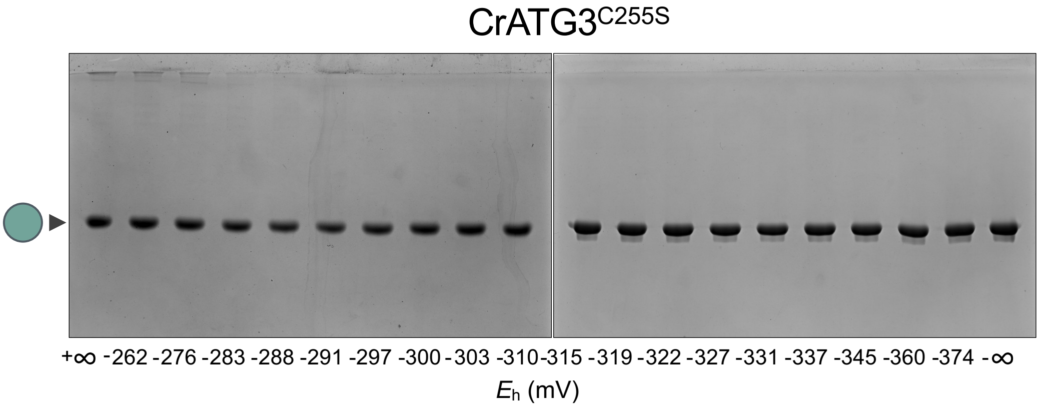

Supplemental Figure S5. Redox titration of Chlamydomonas catalytic Cys-to-Ser CrATG3 mutant protein monomerization. SDS-PAGE of CrATG3^{C255S} lacking Cys255. The CrATG3^{C255S} monomer proportion was monitored after incubation at indicated E_h poised by 20 mM DTT in various dithiol/disulfide ratios (indicated at pH 7.5). All samples were resolved by non-reducing SDS-PAGE gels and then visualized by Coomassie Brilliant Blue. The -∞ and +∞ samples correspond to incubation with only DTT_{red} or DTT_{ox}, respectively. The monomeric CrATG3^{C255S} is indicated with a single green ball.

A

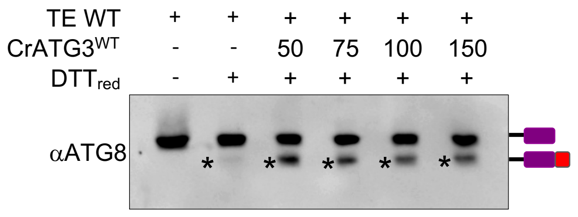

B

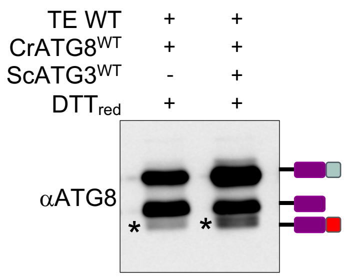

Supplemental Figure S6. Reduced ATG3 mediates Chlamydomonas ATG8 lipidation in cell-free assays. A. Total extracts from Chlamydomonas WT were untreated (-) or treated (+) with DTT_{red} in the absence (-) or presence of increasing concentrations of recombinant $CrATG3^{WT}$ for 60 min at 25ºC. **B.** Total extracts from Saccharomyces WT in a DTT_{red}-containing buffer were incubated with recombinant $CrATG8^{WT}$ in the absence (-) or presence (+) of recombinant $ScATG3^{WT}$ for 60 min at 25ºC. After the assay, proteins were subjected to SDS-PAGE gel and then to western blot analysis with Chlamydomonas anti-ATG8 antibody (αATG8). The different protein versions of CrATG8 (unprocessed, processed and lipidated CrATG8) are indicated on the right. CrATG8-PE is highlighted with an asterisk (on the gel).

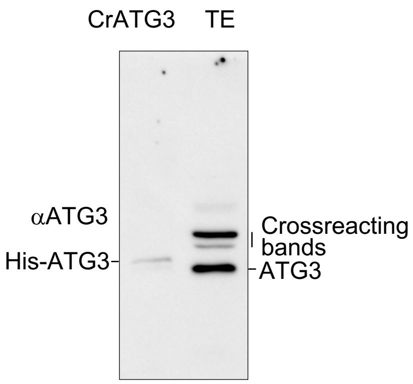

Supplemental Figure S7. CrATG3 antibodies recognize exogenous and endogenous CrATG3 protein. His-tagged ATG3 (5 ng) and total extracts (20 μg) from Chlamydomonas growing exponentially under optimal conditions were subjected to western blot with Chlamydomonas ATG3 antibodies.

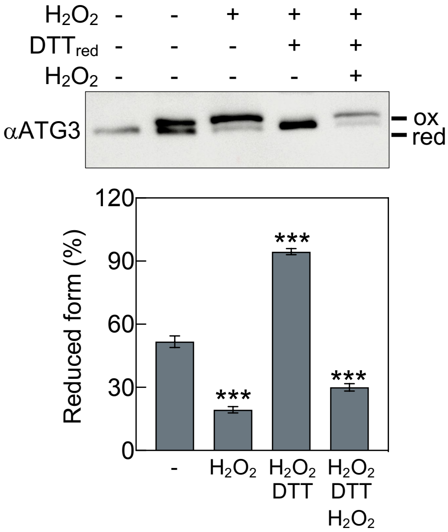

Supplemental Figure S8. Reversibility of Chlamydomonas ATG3 redox state in cell-free total extracts. Total extracts (TE) from Chlamydomonas WT were sequentially incubated (+) with 1 mM H_2O_2 (15 min), 10 mM DTT_{red} (15 min), and then with 50 mM H_2O_2 (15 min), on ice. After, samples were sequentially treated with the alkylating (NEM), reducing (DTT_{red}) and labeling-alkylating ($MM(PEG)_{24}$) agents, and subjected to western blot with anti-CrATG3. red: reduced form; ox: oxidized form. Quantification of reduced ATG3 (%) from at least three independent experiments is shown. Error bars correspond to SD from 3 samples. Asterisks represent significant differences according to one-way ANOVA and Bonferroni's test, ***$P < 0.001$. The statistical analysis is detailed in Supplemental Table S2.

Supplemental Table S1. Proteins used in this study.

Name	Characteristics	Reference
**CrATG8^{WT}**	His-tag WT ATG8 from Chlamydomonas	1
**CrATG3^{WT}**	His-tag WT ATG3 from Chlamydomonas	This study
**CrATG3^{C50S}**	His-tag ATG3 where Cys50 was replaced by Ser from Chlamydomonas	This study
**CrATG3^{C81S}**	His-tag ATG3 where Cys81 was replaced by Ser from Chlamydomonas	This study
**CrATG3^{C255S}**	His-tag ATG3 where the catalytic Cys255 was replaced by Ser from Chlamydomonas	This study
**ScATG3^{WT}**	His-tag WT ATG3 from Saccharomyces	This study
**ScATG4^{C234S}**	His-tag ATG3 where Cys234 was replaced by Ser from Saccharomyces	This study
**CrTRXh1**	His-tag WT TRXh1 from Chlamydomonas	2

Cr: *Chlamydomonas reinhardtii*; **Sc:** *Saccharomyces cerevisiae*.

1. Pérez-Pérez ME, Florencio FJ, Crespo JL (2010). Plant Physiol, 152(4):1874-1888.
2. Goyer A, Decottignies P, Lemaire S, Ruelland E, Issakidis-Bourguet E, Jacquot J-P, Miginiac-Maslow M (1999). FEBS Lett., 444(2-3):165-169.

Supplemental Table S2. Statistical analysis from main figures.

Figure	Experimental condition	Statistical analysis	n	*P* value
6B	αATG3 (8 h control vs 8 h NF)	Two-tailed Student´s *t* test	4	Not significant
6B	αATG3 (24 h control vs 24 h NF)	Two-tailed Student´s *t* test	4	*P*=0.00068 ***
6B	αATG8 (8 h control vs 8 h NF)	Two-tailed Student´s *t* test	4	Not significant
6B	αATG8 (24 h control vs 24 h NF)	Two-tailed Student´s *t* test	4	*P*<0.00001 ***
6B	αATG3 (8 h control vs 8 h Cer)	Two-tailed Student´s *t* test	4	*P*=0.01428 *
6B	αATG3 (24 h control vs 24 h Cer)	Two-tailed Student´s *t* test	4	*P*=0.00075 ***
6B	αATG8 (8 h control vs 8 h Cer)	Two-tailed Student´s *t* test	4	*P*=0.00038 ***
6B	αATG8 (24 h control vs 24 h Cer)	Two-tailed Student´s *t* test	4	*P*<0.00001 ***
6B	αATG3 (8 h control vs 8 h MV)	Two-tailed Student´s *t* test	4	*P*=0.01124 *
6B	αATG3 (24 h control vs 24 h MV)	Two-tailed Student´s *t* test	4	*P*=0.02704 *
6B	αATG8 (8 h control vs 8 h MV)	Two-tailed Student´s *t* test	4	*P*=0.00003 ***
6B	αATG8 (24 h control vs 24 h MV)	Two-tailed Student´s *t* test	4	*P*<0.00001 ***
6B	αATG3 (0 h control vs 3 h HL)	Two-tailed Student´s *t* test	3	*P*=0.03728 *
6B	αATG3 (0 h control vs 6 h HL)	Two-tailed Student´s *t* test	3	*P*=0.03785 *
6B	αATG8 (0 h control vs 3 h HL)	Two-tailed Student´s *t* test	3	*P*=0.00337 **
6B	αATG8 (0 h control vs 6 h HL)	Two-tailed Student´s *t* test	3	*P*=0.00654 **
7C	ATG3 reduced form (%) (0 h vs 5 min H_2O_2)	Two-tailed Student´s *t* test	3	*P*=0.00501 **
7C	ATG3 reduced form (%) (0 h vs 15 min H_2O_2)	Two-tailed Student´s *t* test	3	*P*=0.00110 **
7C	ATG3 reduced form (%) (0 h vs 30 min H_2O_2)	Two-tailed Student´s *t* test	3	*P*=0.00160 **
7C	ATG3 reduced form (%) (0 h vs 30 min DTT_{red})	Two-tailed Student´s *t* test	3	*P*=0.00050 ***
7D	ATG3 reduced form (%) (Control vs DTT_{red})	Two-tailed Student´s *t* test	3	*P*<0.00001 ***
7D	ATG3 reduced form (%) (Control vs DTT_{red}+ H_2O_2)	Two-tailed Student´s *t* test	3	*P*<0.00001 ***
7D	ATG3 reduced form (%) (Control vs DTT_{red}+ H_2O_2+ DTT_{red})	Two-tailed Student´s *t* test	3	*P*<0.00001 ***
7E	ATG3 reduced form (%) (0 h vs 8 h NF)	One-way ANOVA and Bonferroni´s test	3	Not significant
7E	ATG3 reduced form (%) (0 h vs 24 h NF)	One-way ANOVA and Bonferroni´s test	3	*P*=0.00422 **

7E	ATG3 reduced form (%) (0 h vs 48 h NF)	One-way ANOVA and Bonferroni´s test	3	*P*=0.01490 *
7E	ATG3 reduced form (%) (0 h vs 4 h Cer)	One-way ANOVA and Bonferroni´s test	3	Not significant
7E	ATG3 reduced form (%) (0 h vs 8 h Cer)	One-way ANOVA and Bonferroni´s test	3	*P*=0.00070 ***
7E	ATG3 reduced form (%) (0 h vs 24 h Cer)	One-way ANOVA and Bonferroni´s test	3	*P*=0.00011 ***
7E	ATG3 reduced form (%) (0 h vs 4 h MV)	One-way ANOVA and Bonferroni´s test	3	*P*=0.00706 **
7E	ATG3 reduced form (%) (0 h vs 8 h MV)	One-way ANOVA and Bonferroni´s test	3	*P*=0.00110 **
7E	ATG3 reduced form (%) (0 h vs 24 h MV)	One-way ANOVA and Bonferroni´s test	3	*P*=0.00043 ***
7E	ATG3 reduced form (%) (0 h vs 0.5 h HL)	One-way ANOVA and Bonferroni´s test	3	*P*=0.00262 **
7E	ATG3 reduced form (%) (0 h vs 1 h HL)	One-way ANOVA and Bonferroni´s test	3	*P*=0.00634 **
7E	ATG3 reduced form (%) (0 h vs 2 h HL)	One-way ANOVA and Bonferroni´s test	3	Not significant
7E	ATG3 reduced form (%) (0 h vs 3 h HL)	One-way ANOVA and Bonferroni´s test	3	*P*=0.04370 *
7E	ATG3 reduced form (%) (0 h vs 6 h HL)	One-way ANOVA and Bonferroni´s test	3	*P*<0.00001 ***
S8	ATG3 reduced form (%) (Control vs H_2O_2)	Two-tailed Student´s *t* test	3	*P*<0.00001 ***
S8	ATG3 reduced form (%) (Control vs H_2O_2+DTT_{red})	Two-tailed Student´s *t* test	3	*P*<0.00001 ***
S8	ATG3 reduced form (%) (Control vs H_2O_2+DTT_{red}+ H_2O_2)	Two-tailed Student´s *t* test	3	*P*<0.00001 ***

*** *P*<0.001; ** *P*<0.01; **P*<0.05; Not significant ≥0.05

NF: norflurazon; Cer: cerulenin; MV: methyl viologen; HL: high light
